# Impaired extinction of operant cocaine in a genetic mouse model of schizophrenia risk

**DOI:** 10.1007/s00213-023-06386-8

**Published:** 2023-05-26

**Authors:** Rose Chesworth, Gabriela Visini, Tim Karl

**Affiliations:** 1 School of Medicine, Western Sydney University, Campbelltown, NSW Australia; 2 Neuroscience Research Australia, Randwick, NSW Australia

**Keywords:** Conditioned place preference, Self-administration, Locomotor sensitisation, Schizophrenia, Drug use problems, Genetic mouse model

## Abstract

**Background:**

Individuals with schizophrenia have high rates of comorbid substance use problems. One potential explanation for this comorbidity is similar neuropathophysiology in substance use and schizophrenia, which may arise from shared genetic risk factors between the two disorders. Here we investigated if genetic risk for schizophrenia could affect drug reward and reinforcement for cocaine in an established mouse model of genetic risk for schizophrenia, the *neuregulin 1* transmembrane domain heterozygous (*Nrg1 TM* HET) mouse.

**Methods:**

We examined drug-induced locomotor sensitization and conditioned place preference for several cocaine doses (5, 10, 20, 30 mg/kg) in male adult *Nrg1 TM* HET and wild-type-like (WT) littermates. We also investigated intravenous self-administration of and motivation for cocaine (doses 0.1, 0.5, 1 mg/kg/infusion), as well as extinction and cue-induced reinstatement of cocaine. In a follow-up experiment, we examined self-administration, extinction and cue-induced reinstatement of a natural reward, oral sucrose.

**Results:**

Cocaine preference was similar between *Nrg1 TM* HET mice and WT littermates at all doses tested. Locomotor sensitization to cocaine was not affected by *Nrg1* genotype at any dose. Although self-administration and motivation for cocaine was unaffected, extinction of cocaine self-administration was impaired in *Nrg1 TM* HET compared to WT controls, and cue-induced reinstatement was greater in *Nrg1* mutants in the middle of the reinstatement session. Sucrose self-administration and extinction thereof was not affected by genotype, but inactive lever responding was elevated during cue-induced reinstatement for operant sucrose in *Nrg1 TM* HET mice compared to WTs.

**Discussion:**

These results suggest impaired response inhibition for cocaine in *Nrg1 TM* HET mice and suggests *Nrg1* mutation may contribute to behaviours which can limit control over cocaine use.

**Supplementary Information:**

The online version contains supplementary material available at 10.1007/s00213-023-06386-8.

## Introduction

Schizophrenia is a debilitating psychiatric disorder, which is complicated substantially by comorbid drug use problems. Rates of substance use are up to five time higher in individuals with schizophrenia compared to the population at large, and this occurs across a range of drugs of abuse, including nicotine, cannabis, alcohol and psychostimulants such as cocaine and methamphetamine (Dixon 1999; Khokhar et al. 2018; Volkow 2009). Comorbid substance use significantly impairs patient welfare and quality of life, worsening symptoms, decreasing treatment compliance, increasing the risk of psychotic relapse and hospitalisation, and elevating suicide risk (Dixon 1999; Khokhar et al. 2018). It is unclear why individuals with schizophrenia are at greater risk of substance use problems, and why they continue with problematic drug use despite this worsening disease symptoms and progression.

Several theories have been advanced to explain comorbidity between schizophrenia and drug use [reviews: (Green et al. 2007; Khokhar et al. 2018)]. Khokar and colleagues recently proposed a unifying theory, whereby genetic risk for schizophrenia leads to dysfunctional brain reward circuits, which can increase substance use rates in adolescence that triggers schizophrenia development and increases the likelihood of drug use problems in later life, after the development of schizophrenia (Khokhar et al. 2018). Considering the significant role that genetic risk for schizophrenia is hypothesised to play in risk for drug use problems, it is of considerable interest to untangle this relationship.

Investigations into how schizophrenia genetic risk leads to drug abuse susceptibility are still in their infancy. Clinical studies have linked generalised genetic and environmental risk for schizophrenia (e.g. polygenic risk scores) with susceptibility to the abuse of alcohol [(Reginsson et al. 2018) but see conflicting results: (Miller et al. 2019)], nicotine/tobacco (Chen et al. 2016; Miller et al. 2019; Reginsson et al. 2018) and cannabis (Martin et al. 2014; Pasman et al. 2018). A limited number of gene mutations which increase risk for developing schizophrenia appear associated with cannabis abuse [e.g. *Neuregulin 1 (NRG1)* (Han et al. 2012) see also (Aukst Margetic et al. 2019)] and alcohol abuse [e.g. *brain derived neurotrophic factor* (Cheah et al. 2014; Zai et al. 2018), *Neuregulin 1 (NRG1)* (Vaht et al. 2017)]; however, no association has been shown for other genetic variants [e.g. cocaine and amphetamine regulated transcript with nicotine and alcohol abuse (Busto et al. 2010)]. Together, these clinical findings provide some initial support for genes which increase risk for developing schizophrenia also increasing susceptibility for drug use problems. However, examining this research question in clinical cohorts can be confounded by issues such as polydrug use, the polygenetic nature of schizophrenia, and use of antipsychotic medications in patient cohorts. Rodent models provide an excellent tool to investigate causes of comorbidity between substance use and schizophrenia in a controlled manner without putting patients at risk (e.g. symptom intensification, increased risk of relapse). They provide insights into how genetic or environmental factors or combinations of these factors, which can increase the risk of developing schizophrenia, may also increase drug abuse liability and the mechanisms driving increased addiction susceptibility (e.g. increased cue reactivity, susceptibility to relapse, elevated drug reinforcement) (Brown et al. 2012; Menne and Chesworth 2020; Ng et al. 2013). However, to date there has been very limited investigation of how risk genes for schizophrenia affect behaviours relevant for substance use disorders (e.g. Gancarz et al. 2016; Hikida et al. 2020).

*Neuregulin 1* (*NRG1*) is well-established risk gene for schizophrenia [review: (Mostaid et al. 2016)], and a missense mutation in the transmembrane domain of *NRG1* is associated with schizophrenia (Walss-Bass et al. 2006). There is some clinical data linking *NRG1* with substance use disorders (Aukst Margetic et al. 2019; Han et al. 2012; Vaht et al. 2017), but this is yet to be systematically investigated. A mouse model for mutant *NRG1*, the *Nrg1* transmembrane domain heterozygous (*Nrg1 TM* HET) mouse, exhibits behavioural and neurochemical sensitivity to the main psychoactive component of cannabis, ∆^9^-tetrahydrocannabinol (THC) (Boucher et al. 2007a, 2007b; Long et al. 2010, 2013) and other cannabinoid compounds (Boucher et al. 2011), and increased sensitivity to methamphetamine in adolescence (Spencer et al. 2012). Despite *Nrg1 TM* HET mice showing increased susceptibility to the effects of cannabinoids and methamphetamine on schizophrenia-relevant behaviours (e.g. locomotion, social behaviours, sensorimotor gating), behaviours relevant to substance use problems have not been examined in these mice (e.g. drug reward and self-administration, extinction and reinstatement, locomotor sensitization).

Thus, we examined substance use-relevant behaviours for cocaine as well as a natural reinforcer, sucrose in *Nrg1 TM* HET mice. We used cocaine because rates of cocaine use in schizophrenia patients are more than 5 × higher than the general population (Sara et al. 2014; Volkow 2009), and while *Nrg1 TM* HET mice show increased susceptibility to the locomotor stimulating effects of the psychostimulant methamphetamine (Spencer et al. 2012), the response of these mice to cocaine is completely unknown. We used adult male mice in this study, as the schizophrenia-relevant phenotype of *Nrg1* mutants, as well as their susceptibility to cannabinoids is not as pronounced in female mice of this line (Chesworth et al. 2012a; Long et al. 2010). We hypothesised that *Nrg1 TM* HET mice might exhibit increased sensitivity to the rewarding and reinforcing, as well as the locomotor stimulating properties of cocaine. To thoroughly address this question and evaluate if changes to cocaine-relevant behaviours extended to other reinforcers, we examined if changes to cocaine-induced behaviours were also evident using a natural reinforcer, sucrose.

## Materials and methods

### Animals

Male heterozygous *Nrg1* TM^+/−^ mice (*Nrg1 TM* HET) and control *Nrg1* TM^+/+^ (wild-type-like, WT) littermates were bred as described previously (Karl et al. 2007). Mice were bred and housed at Australian BioResources (Moss Vale, Australia). Mice were housed with their dams in litters prior to weaning at PND 21, when they were group-housed in individually ventilated cages (Type Mouse Version 1; Airlaw, Smithfield, Australia; air change: 90–120 times per hour averaged; air speed: 0.12 m/s; passive exhaust ventilation system). Genotypes were confirmed after weaning using tail tip biopsy and polymerase chain reaction amplification (Karl et al. 2007).

At least 2 weeks prior to testing, adult animal animals were transferred to the animal facility at Western Sydney University (School of Medicine, Campbelltown, Australia). Mice were group housed (2–4/cage) in filter top cages (Type 1284B; Tecniplast, Rydalmere, Australia) with corn cob bedding and a wire lid, providing climbing opportunities, a mouse igloo (Bioserv, Frenchtown, USA) and tissues for nesting material. Mice were kept under a 12:12-h light:dark schedule (lights on 9am–9 pm), and food and water were available ad libitum. All animals were 6–7 months of age at the commencement of behavioural testing (average age 7.1 months), in accordance with the age-dependent phenotype observed for this model after 5–6 months of age (Karl et al. 2007). Separate cohorts of experimentally naïve mice were used. Samples sizes are as follows: Experiment 1 (5 mg/kg cocaine CPP): 14 WT, 14 *Nrg1 TM* HET. Experiment 2 (10 mg/kg cocaine CPP): 9 WT, 9 *Nrg1 TM* HET. Experiment 3 (20 mg/kg cocaine CPP): 16 WT, 9 *Nrg1 TM* HET. Experiment 4 (30 mg/kg cocaine CPP): 15 WT, 14 *Nrg1 TM* HET. Experiment 5 (cocaine intravenous self-administration Dose response): 10 WT, 7 *Nrg1 TM* HET. Experiment 6 (cocaine intravenous self-administration extinction and reinstatement): 25 WT, 15 *Nrg1 TM* HET. Experiment 7 (sucrose self-administration): 14 WT, 9 *Nrg1 TM* HET.

Research and animal care procedures were approved by the Western Sydney University Animal Care and Ethics Committee (ACEC) in accordance with the Australian Code of Practice for the Care and Use of Animals for Scientific Purposes (ACEC approval numbers: #A12386, A12135).

### Drugs

Cocaine hydrochloride (National Measurements Institute, ACT, Australia) was dissolved in 0.9% saline. Vehicle injections were 0.9% saline. All injections were given intraperitoneally (i.p.) at a volume of 10 ml/kg body weight as published previously (Chesworth et al. 2013, 2016).

### Conditioned place preference (CPP) - Experiments 1–4

CPP apparatus and methods are identical to those published previously (Chesworth and Karl 2020; Chesworth et al. 2021). The CPP apparatus was a modified open field arena (43.2 × 43.2 × 30.3 cm, Med Associates Inc., VT, USA), with a black Perspex divider separating two equally sized compartments. A small gate (11 × 9 cm) allowed access to both compartments. Wall patterns were used to distinguish the two compartments, with white walls on the left side of the apparatus, and black spots on a white background on the right side of the apparatus (Chesworth and Karl 2020; Chesworth et al. 2021).

An overview of CPP testing is provided in Supplementary Table S1.

Home cages of test mice were transferred into the experimental room at least 30 min prior to testing. On day 1 (habituation), mice were placed randomly in either compartment and allowed free access to the entire apparatus for 30 min. The time spent in each compartment during habituation was used to allocate drug pairings. Mice with a neutral preference (45–55% for either side) were randomly allocated their drug-paired side (unbiased allocation). For mice preferring one side, the drug was paired with the other side (biased allocation) (Chesworth and Karl 2020; Chesworth et al. 2021).

On days 2–4 (conditioning), mice received i.p. injections of saline or cocaine and were immediately confined into one of the two conditioning compartments. Saline conditioning was conducted in the morning (30 min after light phase onset, 0930) and cocaine conditioning (1400) was conducted in the afternoon, with at least 5 h separating the start of vehicle and drug conditioning sessions. Conditioning sessions were 30 min (Chesworth and Karl 2020; Chesworth et al. 2021). This circadian bias was intentional, to ensure cocaine was no longer circulating during saline conditioning sessions. This approach also ensured that saline conditioning, which is less salient that cocaine conditioning, occurred when animals were most alert (i.e. earlier in the light phase), to limit bias toward cocaine-environment learning.

Cocaine doses used were 5 mg/kg, 10 mg/kg, 20 mg/kg and 30 mg/kg, corresponding with Experiments 1 (5 mg/kg), 2 (10 mg/kg), 3 (20 mg/kg) and 4 (30 mg/kg). These doses were chosen because they are frequently used in the literature, and represent low (5 mg/kg), mid (10–20 mg/kg) and high (30 mg/kg) doses (Alaghband et al. 2017; Burgdorf et al. 2017; Fischer et al. 2017; Khroyan et al. 2015; Malvaez et al. 2013; Nguyen et al. 2012).

Locomotor data was also collected throughout these experiments to assess the development of locomotor sensitization for cocaine (i.e. an increase in locomotor activity with repeated cocaine administration). Locomotor data was recorded via horizontal infrared beams and custom software [Activity Monitor, Med Associates Inc.-software settings for the detection of locomotion were box size: 3; ambulatory trigger: 2; resting delay: 1000 ms; resolution: 100 ms (Karl et al. 2007)].

At test, mice were initially placed in the vehicle-paired compartment, and then given free access to the CPP apparatus (i.e. both sides of the compartment accessible as the gate was open). The test sessions were 30 min. A Preference Score was calculated for habituation and test (time in drug zone–time in vehicle zone); a positive preference score indicated a preference for the drug-paired compartment (Chesworth and Karl 2020).

### Intravenous self-administration (IVSA)

Self–administration of intravenous cocaine was assessed using operant chambers (model ENV-022 V, Med Associates, VT, USA) equipped with two levers, one paired with reinforcement (the active lever), the other resulted in no outcome when pressed (the inactive lever). A magazine was located between the two levers, and food pellets were delivered via the magazine during food training. A stimulus light located above the active lever was turned on for 10 s in conjunction with reinforcement (conditioned stimulus, CS). An almond-scented piece of paper (discriminative cue) was placed below the active lever prior to each session. The chambers were housed in sound attenuated boxes and ventilated with fans. During food and cocaine self-administration, mice were tested for 6 consecutive days, and then given 1 day break, before testing resumed. IVSA methods were adapted from previously published work (Chesworth et al. 2013, 2016).

### Sucrose pellet training

All mice in IVSA experiments underwent identical sucrose pellet training prior to surgery. Mice were food restricted during food training and were given 3–4 g chow per mouse per day, keeping mice at 90–95% free feeding weight. Mice were taught to discriminate the active from inactive lever with 10 days of sucrose pellet training, using dustless precision pellets (20 mg, sucrose formulation, Bio-Serv, NJ, USA), to ensure differences in cocaine self-administration were not due to an inability to learn an operant task. Sucrose pellet training sessions were 1 h. Mice were first trained for 1 day to collect sucrose pellets from the magazine (magazine training). Mice were then trained for 2 days to press the active lever for sucrose pellets delivered to the magazine under a fixed ratio 1 (FR1) schedule (single lever training). The inactive lever was not present during single lever training. Mice were then moved to double lever training for 5 days, during which both levers were extended, and mice needed to discriminate between the active and inactive levers. Mice were trained for 1 day on FR1, and then moved to FR2 for 4 days. Mice were returned to *ab libitum* food for at least 3 days prior to surgery.

#### Surgery

After instrumental training, mice were anaesthetised using isoflurane mixed with air (5% induction, 2–3% maintenance) plus meloxicam (3 mg/kg i.p.) and then implanted with indwelling venous cannulae (Mouse Vascular Access Button VAB62BS/25 and mouse jugular catheter C20PU-MJV1451, Instech, PA, USA) as previously described (Chesworth et al. 2013, 2016). Mice were allowed to recover in a heated recovery chamber for 1–2 h post-surgery (Mini-thermacage, Datesand, UK). Mice were treated with neomycin antibiotic diluted in 0.9% saline following surgery and during the 3 days recovery post-surgery, prior to the commencement of behavioural experiments.

### Cocaine self-administration

Mice were connected via a mouse jugular access tether (25G swivel, KVAH62T, Instech, PA, USA). The swivel was connected with 24 inch PU tubing to a 10-ml syringe filled with cocaine solution in an infusion pump. The infusion volume was 20 µl and duration of infusion 2 s. Sessions were terminated if a predetermined maximum number of drug infusions was attained (for 0.1 mg/kg/infusion: 80 infusions; for 0.5 and 1 mg/kg/infusion: 50 infusions), and no drug was administered in the 5 s immediately after each drug infusion. During this period the stimulus light remained active, and any active lever presses were recorded as ‘time out’ responses. All sessions were 1.5 h in length (maximum infusion contingency notwithstanding). Mice were flushed with 0.02 ml heparinised saline daily prior to and immediately after sessions.

For both cohorts, mice were food restricted overnight prior to the first day of cocaine self-administration, to encourage responding. Food restriction was eased over 4 days of acquisition, and mice were kept on ad libitum food for the rest of the experiment.

### Cocaine dose response - Experiment 5

An overview of Experiment 5 is provided in Supplementary Table S2. Mice were tested on three cocaine doses, to determine if the *Nrg1 TM* HET genotype affected cocaine tolerance. Each dose was available for 6 consecutive days; there was 5 days of FR2 and this was followed the next day by 1 day of progressive ratio (PR) responding at each dose (total testing days = 18). Mice were given 1 day off in between doses. PR testing (1.5 h) was used to assess motivation for each dose, where the number of lever presses required for an infusion was progressively increased within each session [PR schedule: 1, 3, 9, 13, 16, 18, 20, 22, 24, 25, 27, 28, 29, 31, 32, 34, 35, 37, 39, 41, 44, 47, 52, 64, 76, 88, 100, 112, 124, 136 (Chesworth et al. 2013, 2016)]. Breakpoint was defined as the final ratio completed within the session (Brown et al. 2009).

Initially, mice were trained using 0.5 mg/kg/infusion cocaine, to ensure acquisition of cocaine responding occurred with an established dose from the literature (Bird et al. 2014). Mice were then tested for responding with a lower dose of cocaine for 6 days (0.1 mg/kg/infusion), and then moved a higher dose of cocaine for 6 days (1 mg/kg/infusion). We used this test order to ensure the highest dose, which could be potentially aversive, did not cause mice to limit their subsequent responding for the next dose.

### Cocaine extinction and reinstatement - Experiment 6

An overview of Experiment 6 is provided in Supplementary Table S3. Following 4 days of acquisition, mice were trained for 10 days on cocaine 0.5 mg/kg/infusion using an FR2 schedule; termed ‘stable FR2’. The average active lever presses on the last 5 days of responding was calculated (‘averaged FR2’), and used to create extinction criteria for each mouse [i.e. 30% of averaged FR2 active lever presses, maintained over 2 consecutive days (Chesworth et al. 2013; Yan et al. 2006)]. Extinction training followed stable FR2, where responses on the active lever were no longer reinforced with a drug infusion. The stimulus light and almond discriminative cue were not present during extinction sessions. There was a change of experimenter at extinction, such that the experimenter conducting extinction and reinstatement was not the same experimenter which conducted sucrose pellet and cocaine self-administration. Extinction sessions ran for 1.5 h and were conducted for up to 10 days. Mice needed to reach extinction criteria to be considered extinguished. If mice did not reach extinction criteria within 10 days, the number of days to reach extinction was assigned as 12, as mice would have needed at least another 2 days to extinguish and this would permit discrimination between mice which met criteria in 10 days vs mice which would have required at least another 2 days to extinguish. If mice met extinction criteria, they did not complete any more extinction sessions and were instead put into cue-induced reinstatement the next day.

For mice which did extinguish, cue-induced reinstatement testing (1.5 h) was conducted the day after extinction criteria was met. For cue-induced reinstatement, the stimulus light and almond discriminative stimulus were reintroduced to the operant chambers, but active lever responses remained unreinforced (Venniro et al. 2016). Active lever responses were not reinforced in the reinstatement session to differentiate responding for drug-associated cues with reacquisition of cocaine self-administration (the latter would occur if cocaine was present at reinstatement) (Khoo et al. 2015). Reinstatement data is presented as cumulative lever presses across the session (Brown et al. 2009).

### Sucrose self-administration - Experiment 7

In Experiment 7, a final cohort of mice was assessed for operant sucrose self-administration, extinction and cue-induced reinstatement, to determine if the extinction deficits observed for cocaine in *Nrg1 TM* HET mice could be a more generalised deficit and thus also be evident when using a natural reinforcer. An overview of Experiment 7 is provided in Supplementary Table S4.

For sucrose self-administration experiments, mice were food restricted 1 day prior to the start of operant training, and then throughout sucrose self-administration experiments, being kept at 90–95% free feeding weight. All sucrose self-administration, extinction and reinstatement sessions were 45 min, as time course analysis of sucrose pellet experiments indicated that lever pressing was greatest in the first 45 min of the test (data not shown). There were no timeouts during sucrose self-administration sessions. Mice were trained to self-administer 10% sucrose which was delivered to the magazine (sucrose administrations are termed ‘deliveries’), with scent and light cues present. Mice were given 1 day of magazine training, then 3 days of single active lever training under FR1. Mice were then moved to double lever training, with 1 day of FR1, to facilitate acquisition of lever discrimination, and 10 days of FR2, to mimic the 10 days of stable cocaine self-administration in Experiment 6. Mice then underwent extinction training for 10 days, and cue-induced reinstatement after meeting extinction criteria, as described above. Unlike cocaine extinction sessions, there was no change of experimenter for sucrose extinction sessions.

### Statistical analysis

Behavioural data were analysed using SPSS Statistics 24 (IBM, NY, USA). One-way analysis of variance (ANOVA) was used to analyse locomotor data from habituation day. For all experiments where we assessed different cocaine doses, we initially analysed all doses together to check for dose-dependent behaviour, and then conducted further ANOVA at each dose to investigate each dose individually. Four, three- and two-way repeated measures (RM) ANOVA with within factors ‘dose’ (IVSA: 0.1, 0.5, 1 mg/kg/infusion cocaine; CPP: 5, 10, 20, 30 mg/kg), ‘days’ (conditioning days), ‘lever’ (active *vs.* inactive lever) and between factor ‘genotype’ (WT vs. *Nrg1 TM* HET) and ‘dose’ (CPP/sensitization 5, 10, 20, 30 mg/kg) were conducted. Where interactions were found, Bonferroni post hoc tests were used to identify group differences at specific time points. To assess the number of days to meet extinction criteria in IVSA, we employed a log-rank Mantel-Cox test, and a Fisher’s exact test was used to determine the proportion of each genotype which extinguished (Chesworth et al. 2013). Data is presented as mean ± standard error of the mean (SEM), and differences were regarded as statistically significant if *p* < 0.05. In figures, post hoc effects of ‘day’ within drug condition are indicated by ‘&’ for WT mice (^&&^*p* < 0.01, ^&&&^*p* < 0.001) and ‘$’ for *Nrg1 TM* HET mice (^$^*p* < 0.05, ^$$^*p* < 0.01). Effects of ‘genotype’ are indicated by ‘*’ (*p < 0.05). RM effects, in the absence of post hocs, are shown by ‘#’ (^#^*p* < 0.05, ^##^*p* < 0.01, ^###^*p* < 0.001).

### Exclusions

One *Nrg1 TM* HET mouse was excluded from the 30 mg/kg CPP test analysis (Experiment 4) because it jumped from the home cage onto the floor immediately prior to testing. One WT mouse was excluded from Experiment 5 (cocaine IVSA) because of health issues identified after the first PR test. One WT mouse from Experiment 5 and one *Nrg1 TM* HET mouse from Experiment 6 were excluded because their catheters became blocked during the experiment, and they were considered not patent.

## Results

### Experiments 1–4: habituation

There was no preference for either compartment at habituation in 3 out of 4 cocaine doses tested (Supplementary Table S5). There was a preference for the right zone in the 10 mg/kg cocaine cohort [‘zone’ *F*(1,16) = 5.7, *p* = 0.03]. However, time course data indicated only a preference for the right zone at particular time points [‘zone’ × ‘time’ interaction, *F*(5,80) = 4.0, *p* = 0.003; Supplementary Figure S1A, B]. Bonferroni post hoc tests demonstrated a significant preference in the first 5-min block of the test in WT mice, and in 2nd last 5 min block in *Nrg1 TM* HET mice only; no other preferences between the zones were detected. This data indicate the apparatus was very largely unbiased.

### Experiments 1–4: locomotor sensitisation

#### Dose analysis

**Fig. 1:**
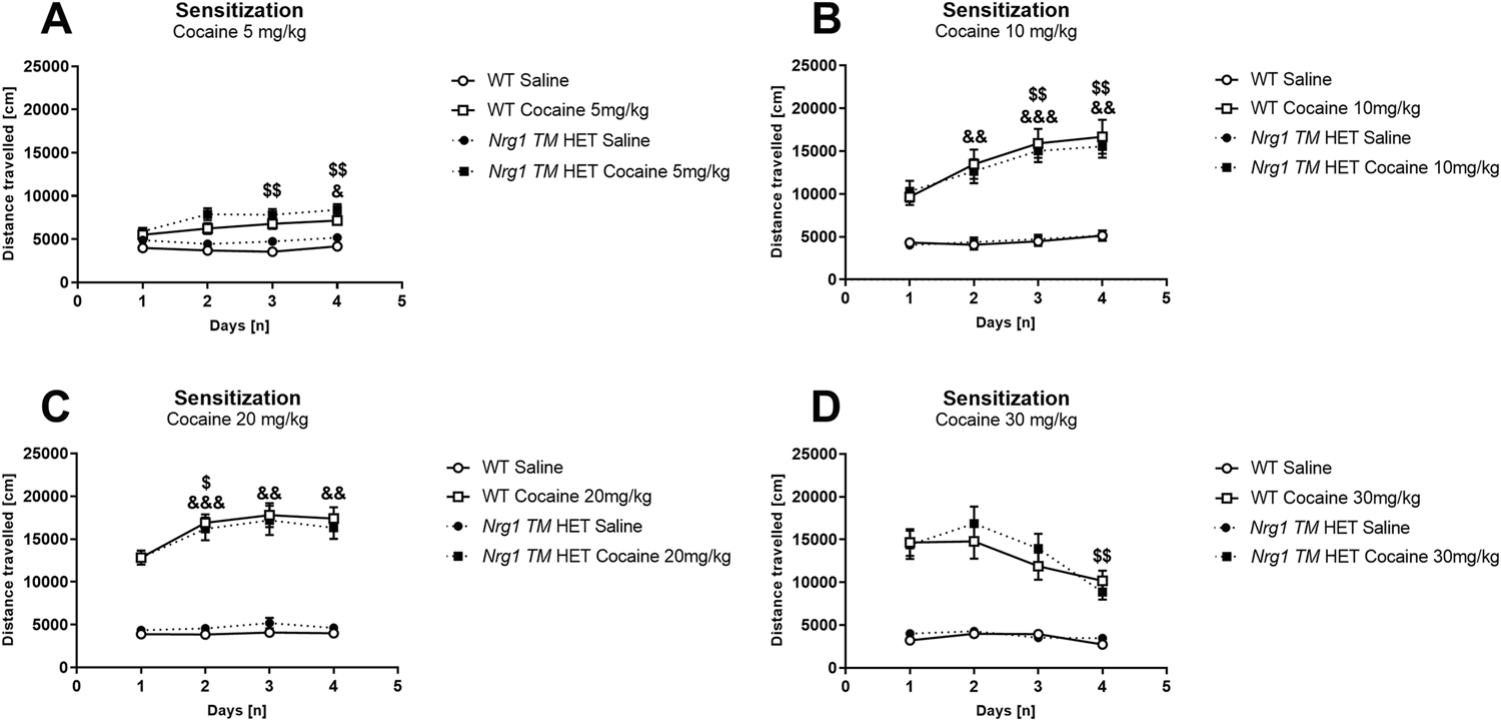
**A**–**D** Locomotor sensitization to cocaine. Distance travelled [cm] in WT and *Nrg1 TM* HET mice following acute saline or cocaine i.p. over 4 consecutive days. Data is presented for **A** 5 mg/kg cocaine, **B** 10 mg/kg cocaine, **C** 20 mg/kg cocaine and **D** 30 mg/kg cocaine. Interactions between ‘days’ × ‘drug’ were present for all doses (all *p*’s < .001). Data analysed by three-way RM ANOVA followed by Bonferroni post hoc tests, and presented as means ± SEM. Within each drug and genotype, significant effects of ‘day’ (vs. the same drug on day 1) are represented by ‘&’ for WT mice (^&&^*p* < .01, ^&&&^*p* < .001) and ‘$’ for *Nrg1 TM* HET mice (^$^*p* < .05, ^$$^*p* < .01)

All cocaine doses increased locomotor activity [four-way RM ANOVA: ‘drug’ *F*(1,91) = 570.1, *p* < 0.001], and higher cocaine doses increased locomotion more than lower doses [‘drug’ × ‘dose’ *F*(3,91) = 38.6, *p* < 0.001] (Fig. 1A–D). Sensitization occurred [‘drug’ × ‘days’ *F*(3,273) = 24.4, *p* < 0.001] and was more pronounced at higher cocaine doses [‘drug’ × ‘days’ × ‘dose’ *F*(9,273) = 14.9, *p* < 0.001]. Genotype had no effect on sensitization (no main effect of ‘genotype’ and no interactions with ‘genotype’: all *p*’s > 0.2). To explore the interaction, we split data by ‘dose’.

All cocaine doses increased locomotion compared to saline (all ‘drug’ *p*’s < 0.001) and both genotypes sensitized to all doses of cocaine (all ‘days’ × ‘drug’ *p*’s < 0.001, no ‘days’ × ‘drug’ × ‘genotype’ interaction for any dose) (Fig. 1A–D). Genotype did not affect locomotor sensitization at any dose (all ‘genotype’ main effects *p*’s > 0.05). Locomotor sensitization was confirmed with post hoc tests, which showed changes to cocaine-induced locomotion in each genotype compared to cocaine day 1 (Fig. 1A–D).

### Experiments 1–4: conditioned place preference

#### Dose analysis

Analysing all doses together, we found an increase in preference score from habituation to test [three-way RM ANOVA: ‘days’ *F*(1,91) = 143.2, *p* < 0.001]. The change in the preference score was more pronounced with higher cocaine doses [‘days’ × ‘dose’ *F*(3,91) = 3.2, *p* = 0.03]; this was not different between the genotypes (no ‘days’ × ‘dose’ × ‘genotype’ interaction, *p* > 0.05). To confirm this, we split data by ‘dose’.

**Fig. 2:**
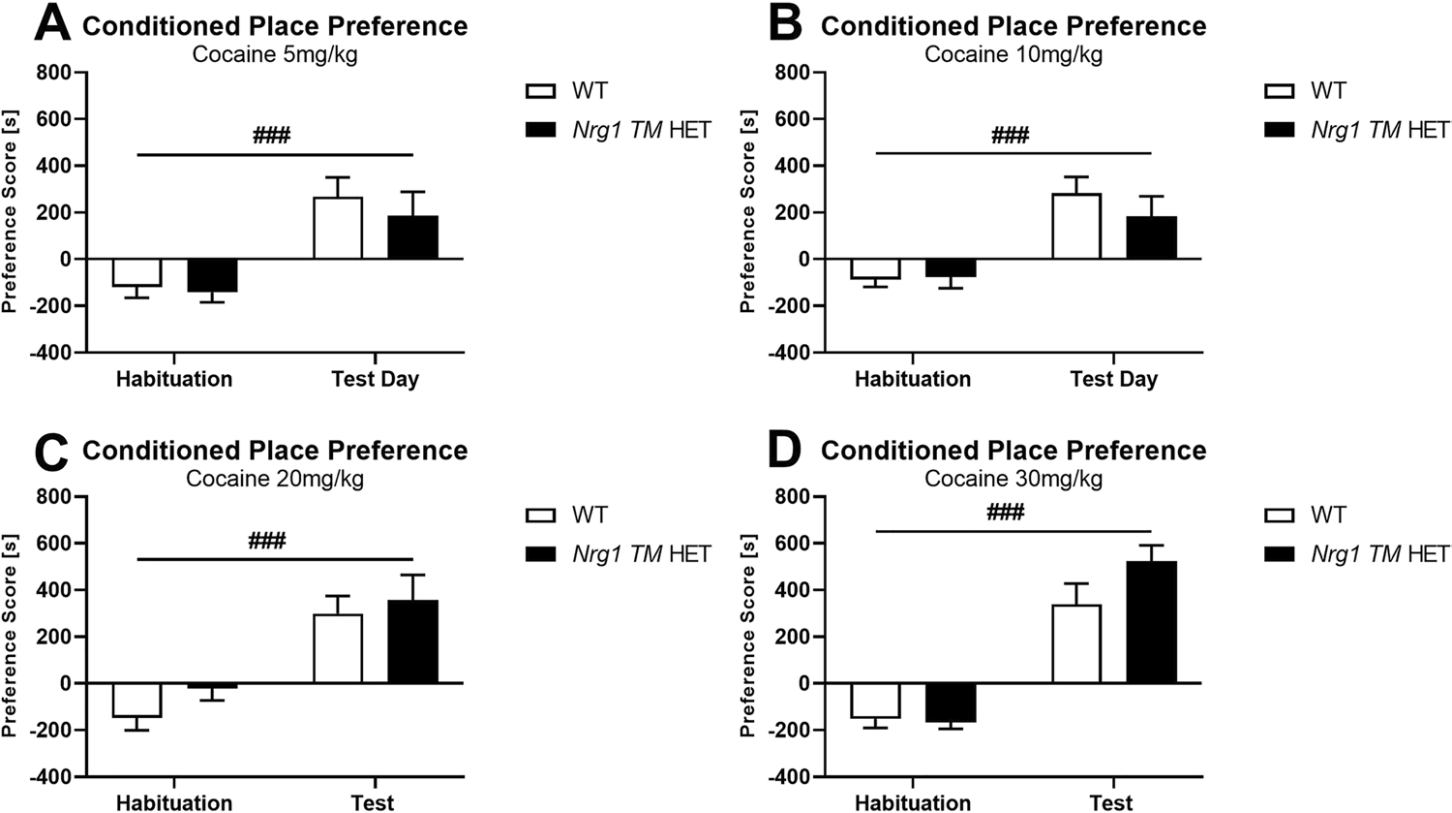
**A**–**D** CPP Preference score at habituation and test. Preference score [s] in WT and Nrg1 TM HET mice at habituation and test. Preference score is defined as (time spent in the cocaine-paired compartment–time spent in the saline-paired compartment). Preference score is presented for each cocaine dose: **A** 5 mg/kg cocaine, **B** 10 mg/kg cocaine, **C** 20 mg/kg cocaine, and **D** 30 mg/kg cocaine. Data analysed by three-way RM ANOVA, and presented as means ± SEM. A ‘days’ × ‘dose’ interaction (*p* = .03) indicates a greater change in preference score with higher cocaine doses. RM effects of ‘day’, showing an increase in preference score at each cocaine dose, are indicated by hash symbols (.^###^*p* < .001)

At all doses tested, mice exhibited an increased preference for the cocaine-paired compartment compared to habituation (‘days’ *p* < 0.001 for all doses) regardless of genotype (*p* > 0.1 for all ‘genotype’ main effects and ‘days’ × ‘genotype’ interactions) (Fig. 2A–D).

### Experiment 5: sucrose pellet self-administration (prior to cocaine self-administration)

Successful acquisition of sucrose pellet self-administration was similar between the genotypes and is presented in Supplementary Figure S2A and 2B.

### Experiment 5: cocaine IVSA—dose response

During the 3 days of initial acquisition, there were no genotype differences in lever responding [three-way RM ANOVA: *F*(1,13) = 0.9, *p* = 0.4] or the number of infusions [*F*(1,13) = 0.2, *p* = 0.7] and no interactions with ‘genotype’ (all *p*’s > 0.05; data not shown). Data during FR2 is presented as the average number of lever presses over the 5 days of FR2 training, as no significant main effects of ‘days’ were detected at each dose.

#### Dose analysis

**Fig. 3:**
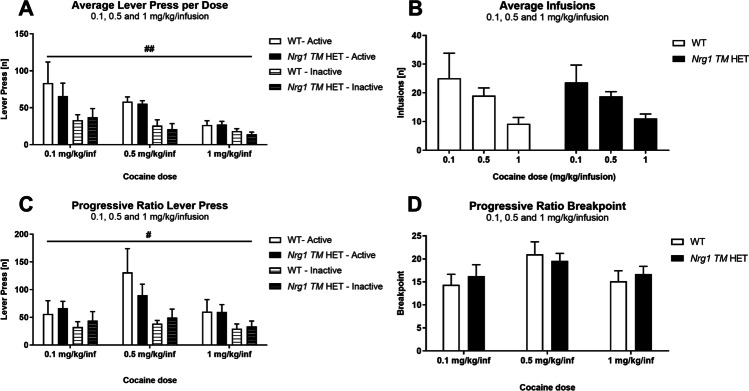
**A**–**C** Intravenous self-administration and motivation for cocaine. **A** Average active and inactive lever pressing [*n*] for cocaine across the 5 days of testing at each dose (0.1, 0.5, 1 mg/kg/infusion), in WT and *Nrg1 TM* HET mice. On average across genotypes, discrimination for the active lever was present for all doses (all *p*’s < .05). **B** Average infusions [*n*] at each cocaine dose (0.1, 0.5, 1 mg/kg/infusion) across the 5 days of testing, in WT and *Nrg1 TM* HET mice. **C** Active and inactive lever pressing [*n*] during progressive ratio testing at each cocaine dose (0.1, 0.5, 1 mg/kg/infusion) in WT and *Nrg1 TM* HET mice. **D** Breakpoint (i.e. last ratio met) for each cocaine dose (0.1, 0.5, 1 mg/kg/infusion) in WT and *Nrg1 TM* HET mice. Data analysed by three- and two-way RM ANOVA, and presented as means ± SEM. A lever type ‘ × ‘dose’ interaction (*p* = .045) in **A** suggests discrimination is higher at lower cocaine doses (0.1 and 0.5 mg/kg/infusion). A lever type ‘ × ‘dose’ interaction (*p* = .01) in **B** suggests discrimination is higher at higher cocaine doses (0.5 and 1 mg/kg/infusion). RM effects of ‘lever type’ are indicated by hash symbols (^#^*p* < .05, ^##^*p* < .01). Abbreviations: Inf: infusion

Lever responding was different between the three doses [three-way RM ANOVA: ‘dose’ *F*(2,26) = 8.8, *p* = 0.001]; examination of Fig. 3A indicates lever responding was highest for the 0.1 mg/kg/infusion and lowest for 1 mg/kg/infusion. There was discrimination for the active lever at all doses [‘lever type’ *F*(1,13) = 20.6, *p* = 0.001] but this was strongest at the 0.1 and 0.5 mg/kg/infusion doses [‘lever type ‘ × ‘dose’ *F*(2,26) = 3.5, *p* = 0.045]. Genotype did not affect lever responding [‘genotype’ *F*(1,13) = 0.1, *p* = 0.7, no ‘genotype’ × ‘dose’ interactions]. We ran individual ANOVA to further analyse genotype and lever effects at each dose.

At each cocaine dose, there was discrimination for the active lever when data were collapsed across both genotypes (all ‘lever type’ *p*’s < 0.02), and lever responding on both active and inactive levers was not different between the genotypes (all ‘genotype’ *p*’s > 0.6, all ‘genotype’ × ‘lever type’ interaction *p*’s > 0.3). There were no genotype differences in inactive lever responding for any dose (0.1 mg/kg/infusion: *F*(1,13) = 0.09, *p* = 0.77; 0.5 mg/kg/infusion: *F*(1,13) = 0.19, *p* = 0.68; 1 mg/kg/infusion: *F*(1,13) = 0.80, *p* = 0.39; additional ANOVA for inactive lever press only).

Both genotypes administered a greater number of infusions at lower cocaine doses than higher doses [‘dose’ *F*(2,26) = 6.6, *p* = 0.005], and the number of infusions made in each genotype was similar across doses [‘genotype’ *F*(1,13) = 0.1, *p* = 0.7; no ‘genotype’ × ‘dose’ interaction *p* = 0.8] (Fig. 3B). For 0.1 mg/kg/infusion cocaine, the average amount of cocaine administered per day was for WT: 0.0753 mg (i.e. 2.2 mg/kg) and for *Nrg1 TM* HET: 0.071 mg (i.e. 2.1 mg/kg). For 0.5 mg/kg/infusion, the average amount of cocaine administered per day was WT: 0.293 mg (i.e. 8.4 mg/kg); *Nrg1 TM* HET: 0.282 mg (i.e. 8.1 mg/kg). For 1 mg/kg/infusion, the average amount of cocaine administered per day was WT: 0.276 mg (i.e. 7.8 mg/kg); *Nrg1 TM* HET: 0.345 mg (i.e. 9.9 mg/kg).

### Experiment 5: cocaine IVSA—progressive ratio

#### Dose analysis

Lever responding under a PR schedule changed across doses [three-way RM ANOVA: ‘dose’ *F*(2,26) = 12.7, *p* < 0.001], but was not different between genotypes [‘genotype’ *F*(1,13) = 0.01, *p* = 0.9; no ‘genotype’ × ‘dose’ interactions]. There was discrimination for the active lever [‘lever type’ *F*(1,13) = 8.5, *p* = 0.01], which was stronger at some doses than others [‘lever type’ × ‘dose’ *F*(2,26) = 5.3, *p* = 0.01] (see Fig. 3C—e.g. 0.5 and 1 mg/kg/infusion; see statistics below). To examine genotype and lever type effects at each dose, we conducted two-way ANOVA for each dose.

There were no genotype differences in PR lever presses at any dose tested (all ‘genotype’ *p*’s > 0.6, Fig. 3C). Discrimination for the active lever was evident at 0.5 and 1 m/kg/infusion PR doses (‘lever type’ *p*’s < 0.02); but not at 0.1 mg/kg/infusion [*F*(1,13) = 3.9, *p* = 0.07]. Additional ANOVA for inactive lever presses demonstrated no genotype differences for any dose (0.1 mg/kg/infusion: *F*(1,13) = 0.38, *p* = 0.55; 0.5 mg/kg/infusion: *F*(1,13) = 0.49, *p* = 0.50; 1 mg/kg/infusion: *F*(1,13) = 0.09, *p* = 0.78). Breakpoint data found no genotype differences for any dose (all ‘genotype’ *p*’s > 0.05; Fig. 3D).

### Experiment 6: cocaine IVSA—extinction and cue-induced reinstatement

**Fig. 4:**
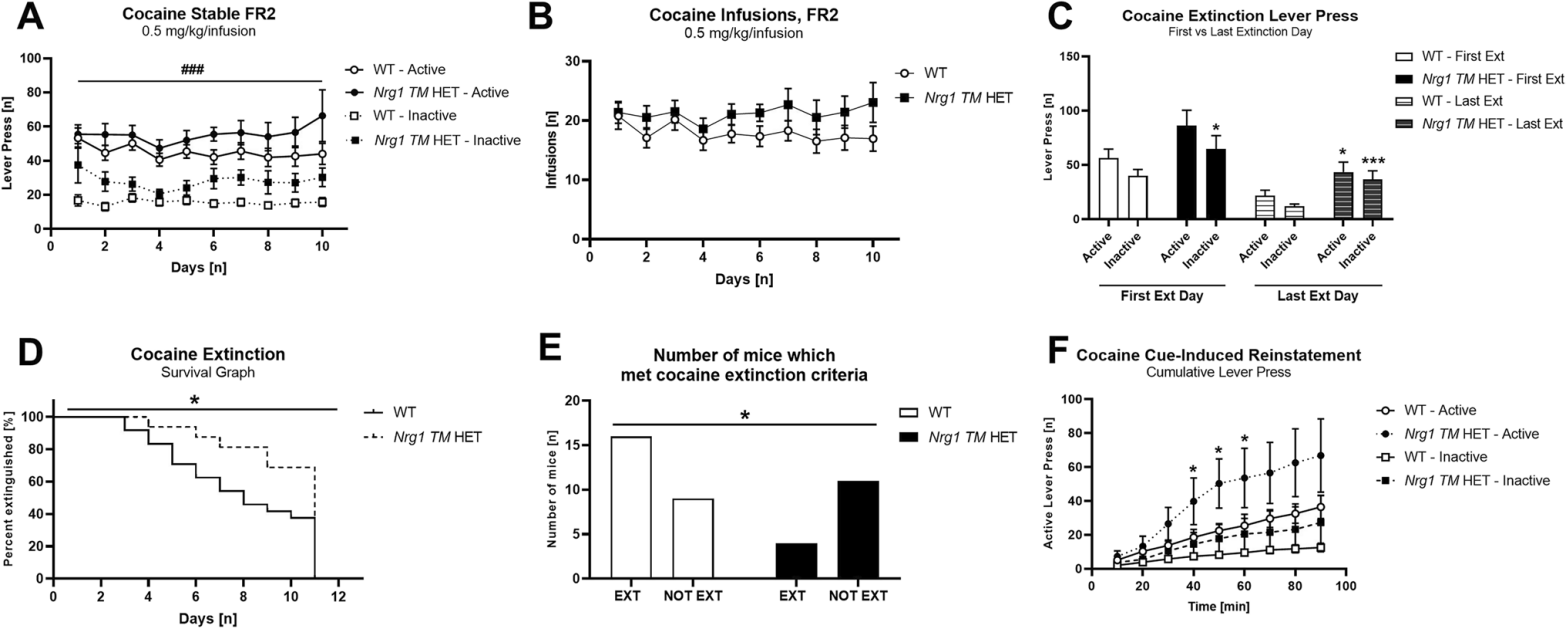
**A**–**F** Self-administration, extinction and cue-induced reinstatement of instrumental cocaine. **A** Active and inactive lever press [*n*] for 0.5 mg/kg/infusion cocaine in WT and *Nrg1 TM* HET mice, over 10 days of self-administration. Both genotypes showed discrimination for the active lever (*p* < .05). **B** Infusions [*n*] of 0.5 mg/kg/infusion cocaine in WT and *Nrg1 TM* HET mice over 10 days of self-administration. **C** Active and inactive lever press [*n*] on the first and final day of cocaine extinction for WT and *Nrg1 TM* HET mice. **D** Survival curve for WT and *Nrg1 TM* HET mice during extinction of cocaine self-administration. **E** Number of WT and *Nrg1 TM* HET mice which reached extinction criteria and did not reach extinction criteria. **F** Cumulative lever pressing [*n*] during cue-induced reinstatement in WT and *Nrg1 TM* HET mice. Data presented as means ± SEM in **A**, **B**, **C** and **F**, and analysed with three- or two-way RM ANOVA. Data in **D** analysed with log-rank Mantel-Cox test. Data in** E** analysed with Fisher’s exact test. ‘Genotype’ main effects in **D** and **E**, and post hoc effects in **C** and **F** indicated by asterisks (*p < .05). RM effects of ‘lever type’ are indicated by hash symbols (^###^*p* < .001). Abbreviations: Ext: mice which met extinction criteria; Not Ext: mice which did not meet extinction criteria

Mice were trained to self-administer 0.5 mg/kg/infusion cocaine under an FR2 schedule for 10 days prior to extinction training. During this period (i.e. stable FR2), both genotypes showed discrimination for the active lever [‘lever type’ *F*(1,37) = 74.0, *p* < 0.001; no ‘genotype’ × ‘lever type’ interaction] and responding was stable across days [‘days’ *F*(9,333) = 1.9, *p* = 0.06]. Overall lever responding was higher in *Nrg1 TM* HET mice than WTs [‘genotype’ *F*(1,37) = 6.5, *p* = 0.02], and examination of Fig. 4A suggests this is due to higher inactive lever pressing in *Nrg1 TM* HET mice. Subsequent analyses of inactive lever pressing only demonstrates elevated inactive lever pressing throughout stable FR2 [‘genotype’ *F*(1,37) = 11.3, *p* = 0.002]; this effect was consistent across stable FR2 (no interaction with ‘days’ *p* > 0.2). There were no genotype differences in active lever presses (*p* = 0.14).

Average active and inactive lever presses across the final 5 days of self-administration (average FR2) were higher in *Nrg1 TM* HET mice than WTs [‘genotype’ *F*(1,37) = 6.4, *p* = 0.02] (Table 1), with similar discrimination for the active lever in both genotypes [‘lever type’ *F*(1,37) = 46.7, *p* < 0.001] (Table 1). *Nrg1 TM* HET mice pressed more on the inactive lever than WT mice [ANOVA for inactive lever press, ‘genotype’ *F*(1,37) = 9.2, *p* = 0.004]; this genotype difference was not evident for active lever press (*p* = 0.11). Importantly, despite higher lever responding in *Nrg1 TM H*ET mice, cocaine infusions were similar between the genotypes during FR2 training [‘genotype’ *F*(1,37) = 1.8, *p* = 0.2] (Fig. 4B). The average amount of cocaine administered per day was WT: 0.268 mg (i.e. 7.7 mg/kg); *Nrg1 TM* HET: 0.302 mg (i.e. 8.6 mg/kg).

**Table 1:** Animal numbers per experiment

Experiment number	Experiment name	WT	*Nrg1 TM* HET
1	CPP 5 mg/kg cocaine	14	14
2	CPP 10 mg/kg cocaine	9	9
3	CPP 20 mg/kg cocaine	16	9
4	CPP 30 mg/kg cocaine	15	14
5	Cocaine IVSA dose response	10	7
6	Cocaine IVSA extinction + reinstatement	25	15
7	Sucrose self-administration	14	9

*Abbreviations*: *CPP* conditioned place preference, *IVSA* intravenous self-administration

Mice were then put into extinction. We confirmed that extinction occurred by comparing lever pressing on the first day of extinction to the day when mice met extinction criteria (for mice which extinguished, this would be the lever presses on the day they extinguished, and for the mice which didn’t extinguish, this was the lever presses on day 10). Mice reduced their lever pressing across days [‘days’ *F*(1,37) = 41.5, *p* < 0.001], and *Nrg1 TM* HET mice exhibited elevated lever pressing compared to WT mice [‘genotype’ *F*(1,37) = 7.9, *p* = 0.008]. Responding decreased across days on the active lever more than the inactive lever in both genotypes [‘lever type’ × ‘days’ *F*(1,37) = 5.9, *p* = 0.02] and post hoc tests indicate that inactive lever responding was higher in *Nrg1 TM* HET mice than WT mice at the beginning and end of extinction, while active lever responding was only higher in *Nrg1* mutants on the final day of extinction (Fig. 4C).

Despite this, not all mice met extinction criteria, and we detected an extinction deficit in *Nrg1 TM* HET mice, as these mice took longer to reach extinction criteria than WT mice (log-rank Mantel-Cox test, *χ*^2^ = 4.56, df = 1, *p* = 0.03) (Fig. 4D). In addition, fewer *Nrg1 TM* HET mice met extinction criteria than WT mice-27% of *Nrg1* mutant mice met extinction criteria, vs 64% WT mice (Fisher’s exact test, *p* < 0.05) (Fig. 4E). Comparing the timecourse of lever pressing in mice which did extinguish on the first and the final days of extinction, there was a decrease in lever pressing across days [‘days’ *F*(1,18) = 34.8, *p* < 0.001], and *Nrg1 TM* HET mice pressed more during the first half of the session across both extinction days [‘genotype’ × ‘minutes’ *F*(8,144) = 2.5, *p* = 0.01]. Bonferroni post hoc tests confirm elevated active and inactive lever responding in *Nrg1* mutant mice on the first and final day of extinction (Supplementary Figure S3A, 3B).

During cue-induced reinstatement, while there were no overall genotype differences in lever responding [‘genotype’ *F*(1,18) = 4.4, *p* = 0.05], active lever responding was greater in *Nrg1* mutants than WTs as the test progressed [‘lever type’ × ‘time’ × ‘genotype’ [*F*(8,144) = 2.1, *p* = 0.04; ‘time’ × ‘genotype’ *F*(8,144) = 4.0, *p* < 0.001] (Fig. 4F). Bonferroni post hoc tests indicate active lever presses were greater in *Nrg1 TM* HET mice compared to WTs at 40–60 min of the reinstatement session (Fig. 4F).

Individuals analyses of active and inactive lever presses separately responding confirmed our interpretation: active lever responding was greater in *Nrg1* mutants compared to WT mice [‘genotype’ *F*(1,18) = 4.6, *p* = 0.047], and this genotype effect was more prominent as the test progressed [‘genotype’ × ‘time’ F(8,144) = 3.7, *p* < 0.001]. While inactive lever pressing was not elevated in *Nrg1* mutants uniformly throughout the reinstatement session [‘genotype’ *F*(1,18) = 2.6, *p* = 0.1], a ‘genotype’ × ‘time’ interaction suggests *Nrg1 TM* HET mice pressed more on the inactive lever as the test progressed [‘genotype’ × ‘time’ *F*(8,144) = 2.3, *p* = 0.02].

To control for differences in lever responding during extinction in *Nrg1 TM* HET mice, we compared total lever pressing during reinstatement to the final day of extinction. There was a strong trend for an effect of genotype [*F*(1,18) = 4.3, *p* = 0.05], suggesting *Nrg1 TM* HET mice tended to show higher reinstatement lever responding when controlling for extinction lever pressing (Supplementary Figure S4A). Drug-associated cues increased responding in both genotypes at reinstatement, particularly on the active lever [‘days’ *F*(1,18) = 21.3, *p* < 0.001; ‘days’ × ‘lever type’ *F*(1,18) = 27.1, *p* < 0.001].

### Experiment 7: sucrose self-administration

**Fig. 5:**
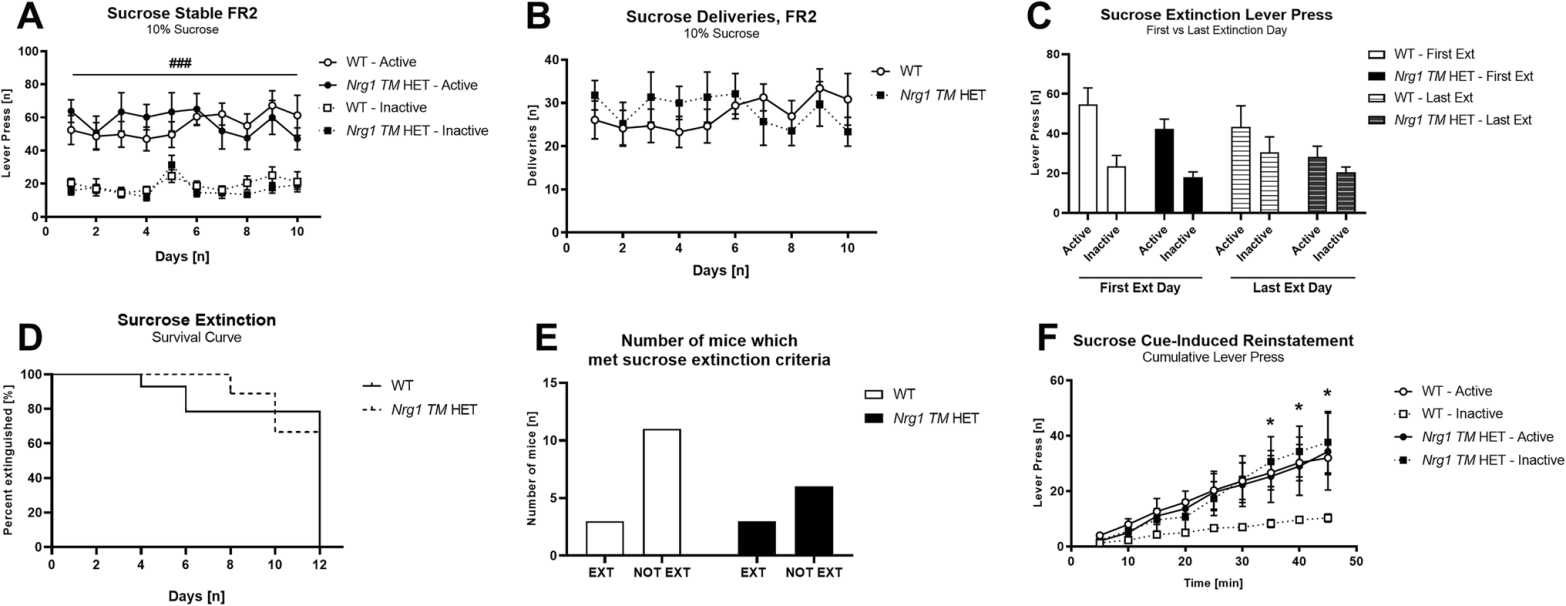
**A**–**F** Self-administration, extinction and cue-induced reinstatement of instrumental sucrose.** A** Active and inactive lever press [*n*] for 10% sucrose in WT and *Nrg1 TM* HET mice, over 10 days of FR2 self-administration. Both genotypes showed discrimination for the active lever (*p* < .05). **B** Sucrose rewards [*n*] in WT and *Nrg1 TM* HET mice over 10 days of self-administration. **C** Active and inactive lever press [*n*] on the first and final day of sucrose extinction for WT and *Nrg1 TM* HET mice. **D** Survival curve for WT and *Nrg1 TM* HET mice during extinction of sucrose self-administration. **E** Number of WT and *Nrg1 TM* HET mice which reached extinction criteria and did not reach extinction criteria. **F** Cumulative lever pressing [*n*] during cue-induced reinstatement for sucrose in WT and *Nrg1 TM* HET mice. Data presented as means + SEM in **A**, **B**, **C** and **D**, and analysed with three- or two-way RM ANOVA. Data in **D** analysed with log-rank Mantel-Cox test. Data in** E** analysed with Fisher’s exact test. RM effects of ‘lever type’ are indicated by hash symbols (^###^*p* < .001). Bonferroni post hoc effects of ‘genotype’ on both active and inactive levers in **F** indicated by asterisks (*p < .05). Abbreviations: Ext: mice which met extinction criteria; Not Ext: mice which did not meet extinction criteria

Both genotypes stably self-administered sucrose during the 10 days of FR2 (Fig. 5A) [no main effect of ‘days’ *F*(9,189) = 1.6, *p* = 0.1 or ‘genotype’ *F*(1,21) = 0.001, *p* = 0.9], and discriminated for the active lever over the inactive lever [‘lever type’ *F*(1,21) = 89.6, *p* < 0.001] (no genotype differences in single lever FR1, data not shown). While there was a ‘genotype’ × ‘lever type’ × ‘days’ interaction [*F*(9,189) = 2.9, *p* = 0.003], follow up post hoc tests did not show any genotype differences in active or inactive lever presses (Fig. 5A). Analysis of active and inactive lever presses using separate ANOVA found no effects of ‘genotype’ on active [*F*(1,21) = 0.1,* p* = 0.9] or inactive lever pressing [*F*(1,21) = 0.4,* p* = 0.6], confirmed this interpretation.

Extinction criteria were created based on the final 5 days of sucrose self-administration (stable FR2). Sucrose self-administration was similar between the genotypes in the last 5 days of training [no main effect of ‘genotype’ *F*(1,21) = 0.6, *p* = 0.4, no interaction] and discrimination for the active lever was evident in both groups [‘lever type’ [*F*(1,21) = 78.7, *p* < 0.001, no interaction with ‘genotype’] (Table 2). Sucrose deliveries were also similar between the genotypes during FR2 training (no main effect of ‘genotype’ *F*(1,21) = 0.1, *p* = 0.8 or ‘days’ *F*(9,189) = 1.7, *p* = 0.1; no interaction] (Fig. 5B).

**Table 2:** Average FR2 responding for 0.5 mg/kg infusion cocaine (Experiment 6) and 10% sucrose (Experiment 7). Average active and inactive lever presses over the last 5 days of stable FR2 training in WT and* Nrg1 TM* HET mice

Experiment number	Experiment number + name	WT	*Nrg1 TM* HET
6	Cocaine 0.5 mg/kg average active lever press	44.5 ± 4.6	57.9 ± 7.2
6	Cocaine 0.5 mg/kg average inactive lever press	15.5 ± 1.8	28.6 ± 4.8
7	10% sucrose average active lever press	61.3 ± 7.1	54.3 ± 8.3
7	10% sucrose average inactive lever press	20.3 ± 3.8	15.8 ± 2.2

*Abbreviations*: *FR2* fixed ratio 2

Comparing the first and last extinction days, we saw no overall reduction in lever pressing during sucrose extinction [‘days’ *F*(1,21) = 1.7, *p* = 0.2], although a ‘lever type’ × ‘days’ interaction suggests active lever responding decreased and inactive lever responding did not (Fig. 5C). *Nrg1 TM* HET mice did not make more lever responses that WTs during extinction [‘genotype’ *F*(1,21) = 1.4, *p* = 0.3], and Bonferroni post hoc tests found no genotype differences at any time point (Fig. 5C).

There were no genotype differences in the latency to reach extinction criteria (log-rank Mantel-Cox test, *χ*^2^ = 0.18, df = 1, *p* = 0.67) (Fig. 5D). Three mice per genotype reached extinction criteria and the proportion in each genotype was similar (21% of WT, 33% of *Nrg1 TM* HET; Fisher’s exact test, *p* = 0.64) (Fig. 5E). When we analysed timecourse data for lever pressing on the first and last days of extinction in mice which did extinguish, there were no genotype differences in lever responding [‘genotype’ *F*(1,4) = 1.5, *p* = 0.3; no interactions with ‘genotype’] (Supplementary Figure S3C, 3D), and all mice reduced their lever responding across days [‘days’ [*F*(1,4) = 45.4, *p* < 0.001], more so on the active lever [‘days’ × ‘lever type’ *F*(1,4) = 33.1, *p* < 0.001].

At cue-induced reinstatement, *Nrg1 TM* HET pressed more on both active and inactive levers as the test progressed [‘time’ × ‘genotype’ interaction *F*(8,32) = 12.8, *p* < 0.001; no main effect of ‘genotype’ *F*(1,4) = 4.2,* p* = 0.1] (Fig. 5F). Bonferroni post hoc tests indicated elevated lever responding on both levers was evident in *Nrg1* mutant mice compared to WT controls in the last 30 min of the session. Furthering this, individual ANOVA for active and inactive lever pressing during reinstatement found no genotype differences on active lever presses [‘genotype’ *F*(1,4) = 0.1, *p* = 0.9], but a ‘time’ × ‘genotype’ interaction for inactive lever presses suggests elevated inactive lever pressing in *Nrg1 TM* HET mice as the reinstatement test progressed [*F*(8,32) = 7.8, *p* < 0.001; no ‘genotype’ main effect *p* = 0.1].

When comparing total lever pressing during reinstatement to the final extinction day, *Nrg1 TM* HET mice pressed more on both levers across both testing days [‘genotype’ *F*(1,4) = 9.7, *p* = 0.04] (Supplementary Figure S4B). Both genotypes increased their responding at cue-induced reinstatement [‘days’ *F*(1,4) = 42.9,* p* = 0.003].

## Discussion

We found impaired extinction of operant cocaine but not sucrose in *Nrg1 TM* HET mice. Responding on both active and inactive levers was greater in *Nrg1 TM* HET mice during cocaine cue-induced reinstatement, compared to WT controls. *Nrg1* mutant mice also exhibited higher levels of inactive lever pressing during operant cocaine self-administration, extinction and cue-induced reinstatement, as well as during sucrose cue-induced reinstatement. Despite this, *Nrg1 TM* HET mice did not show altered cocaine or sucrose self-administration, nor were there genotype differences in cocaine reward, locomotor sensitization to cocaine, or cocaine motivation. This suggests *Nrg1 TM* HET mice may have deficits in response inhibition in the absence of cocaine, despite similar cocaine and sucrose reward and reinforcement.

We detected operant extinction deficits in *Nrg1 TM* HET mice for cocaine across several measures: *Nrg1* mutant mice made more lever responses during cocaine extinction, took longer to meet cocaine extinction criteria, and a greater proportion of *Nrg1* mutant mice did not meet cocaine extinction criteria. We also observed higher cumulative active and inactive lever responding during the middle to later part of cocaine cue-induced reinstatement. While the reinstatement data suggests elevated responding to cocaine-associated cues in *Nrg1* mutant mice, this finding may also be affected by extinction deficits in *Nrg1* mutant mice. Indeed, cue-induced responding under extinction conditions occurs early in a session, after which animals learn that drug cues do not result in drug delivery. As a consequence, mice reduce their responding and thereby demonstrate within session extinction. Thus, elevated responding which only occurs *later* in the cocaine cue reinstatement session may represent impaired within-session extinction in *Nrg1* mutant mice. Interestingly, extinction deficits were not observed for operant sucrose, and active lever responding was not elevated during sucrose cue reinstatement in *Nrg1* mutant mice, suggesting extinction impairments may be cocaine-specific. Linking this to clinical data, deficits in response inhibition are evident in individuals with schizophrenia and are linked to cognitive impairment (Gotra et al. 2020); however, it is unknown if response inhibition is linked to genetic risk for schizophrenia. Our data suggest that impaired response inhibition can extend to drug reinforcers such as cocaine in the presence of *Nrg1* mutation.

There has been limited investigation of how mutations in *Nrg1* *TM *may cause dysregulation of circuits involved in inhibitory control during extinction. *Nrg1 TM* HET mice show elevated cortical gamma frequency power and reductions in sensory-driven gamma responses, potentially representing increased ‘noise’ in neural networks (Long et al. 2015), which may impair cognitive processes which drive extinction learning. Gamma oscillations are linked with working memory abilities necessary for within-session extinction (Yamamoto et al. 2014), and elevated hippocampal network oscillations can underpin resistance to extinction (Caliskan and Stork 2019). It is possible that the *Nrg1 TM* HET mutation regulates hippocampal and/or cortical extinction processes which are relevant for cocaine extinction learning.

We did not observe extinction impairments for operant sucrose, and in fact, very few mice in the operant sucrose experiment reached extinction criteria. While it is possible *Nrg1 TM* HET mutation specifically affects operant cocaine extinction, one methodological difference should be considered before drawing this conclusion. Unlike cocaine extinction, there was no change of experimenter at extinction for sucrose, and an experimenter may serve as a contextual cue which signals drug availability. Indeed, rodents are sensitive to changes in experimenter and this can affect subsequent behaviour (Davis et al. 1997; van Driel and Talling 2005)—see also (Sorge et al. 2014)). Thus, the protocols were not identical due to factors outside of our control, and it is unclear if we may have observed a greater proportion of animals extinguishing, and/or observed extinction deficits in *Nrg1* mutants for operant sucrose with a change of experimenter at extinction. Considering Nrg1 and its main receptor ErbB4 are linked to fear extinction deficits (Cao et al. 2021; Skirzewski et al. 2020), we cannot rule out that *Nrg1 TM* HET mutation may affect extinction processes for both cocaine and natural reinforcers.

We observed higher levels of inactive lever responding in *Nrg1 TM* HET mice compared to WT controls in Experiments 6 and 7. Higher inactive lever responding may be linked to the locomotor hyperactivity phenotype of *Nrg1 TM* HET mice (Karl et al. 2007; Visini et al. 2022); however, this explanation is unlikely as hyperactivity would theoretically increase responding on both levers, not just the inactive lever. Another possibility is that *Nrg1* mutant mice incorrectly overgeneralise active lever contingencies to the inactive lever. Generalisation of active lever responding to a novel lever can occur when training for both levers occurs in the same context (Bernal-Gamboa et al. 2018), and when stimuli are perceptually similar (Bergstrom 2020). While generalisation of learning can be beneficial (e.g. prior stimulus associations can predict reinforcement from a novel stimulus), *overgeneralisation* can be maladaptive (Dunsmoor and Paz 2015), and may be considered a form of cognitive impairment, representing an inability to engage in sufficient pattern separation to distinguish two distinct stimuli (Abumaria et al. 2013). *Nrg1 TM* HET mice exhibit cognitive deficits in novel object recognition and recall of contextual fear conditioning (Duffy et al. 2010), while in clinical populations, *NRG1* SNPs and serum NRG1β1 concentration are linked with poorer cognitive ability in individuals with schizophrenia (Cho et al. 2015; Yang et al. 2021). Thus, it is possible that elevated inactive lever pressing may reflect cognitive impairment in *Nrg1* mutants.

We found no genotype differences in cocaine reward in CPP or cocaine reinforcement in IVSA. We hypothesised that cocaine reward and reinforcement may be enhanced by *Nrg1 TM* HET mutation, as *Nrg1 TM* HET mice exhibit reduced striatal dopamine D_2_ receptor density (Newell et al. 2013), and enhanced striatal D_2_ receptor expression can reduce cocaine self-administration in rats [(Thanos et al. 2008) see also (Nader et al. 2006)]. However, across a dose range in both CPP and IVSA, we found that mutant *Nrg1* does not affect acquisition and expression of cocaine reward or cocaine operant learning. It is possible that the degree of striatal D_2_ receptor reduction in *Nrg1* mutants was insufficient to alter cocaine reward or reinforcement. Indeed, other studies have shown a > 40% increase in striatal D_2_ receptors reduces cocaine self-administration in rats (Thanos et al. 2008), and striatal D_2_ receptors in *Nrg1 TM* HET mice are only reduced by ~ 22% (Newell et al. 2013). Alternatively, it is possible that stress may enhance cocaine reward or reinforcement in *Nrg1 TM* HET mice. *Nrg1 TM* HET mice are more susceptible than WT controls to effects of stress on schizophrenia-relevant behaviours (Chesworth et al. 2012b), and individuals carrying an *NRG1* variant who experience stressful life events are more likely to use alcohol, tobacco and illicit drugs than to individuals who do not carry the *NRG1* variant (Vaht et al. 2017). Thus, a gene × environment interaction may be necessary to demonstrate effects of *Nrg1 TM* HET mutation on cocaine reward or reinforcement.

Cocaine-induced locomotion and the development of cocaine locomotor sensitization was intact in *Nrg1 TM* HET mice. Interestingly, we observed a reduction in locomotor activity with repeated 30 mg/kg cocaine in both genotypes, which may indicate an earlier peak in locomotor activity and an increase in stereotyped behaviour with repeated cocaine (Crittenden et al. 2021). It was somewhat surprising that no genotype differences in cocaine locomotor sensitization were detected, as stereotyped behaviours following repeated amphetamine are linked to genes regulated by *Nrg1* (Crittenden et al. 2021), and adolescent *Nrg1* mutant mice show a dose-dependent increase in methamphetamine-induced locomotion (Spencer et al. 2012), although no genotype differences are evident for amphetamine-induced locomotion in adulthood (van den Buuse et al. 2009). It is possible that differences in cocaine sensitization may be dependent on age; indeed, our laboratory has found that *Nrg1 TM* HET mice are differently affected by cannabinoids such as THC and cannabidiol during adolescence compared to adulthood (Boucher et al. 2007a; Long et al. 2013, 2012; Visini et al. 2022). Future research can investigate if *Nrg1 TM* HET mice show altered susceptibility to cocaine locomotor sensitization in adolescence.

We observed some slight differences between Experiments 5 and 6, whereby *Nrg1 TM* HET mice exhibited elevated lever responding for 0.5 mg/kg/infusion cocaine (collapsed across both levers) in Experiment 6, which was not present in Experiment 5. It is possible this is due to the duration of training under the same dose. In Experiment 5, mice were kept on 0.5 mg/kg/infusion for 5 days (4 × FR2 and 1xPR), whereas in Experiment 6, they were kept at this dose for 10 days. Importantly, examination of Fig. 4A suggests that genotype differences become more prominent after the first 5 days. It is possible that if we had kept mice in Experiment 5 at 0.5 mg/kg/infusion for longer that they may have shown the genotype differences observed in Experiment 6.

There were some limitations to the present study. First, the duration of cocaine or sucrose self-administration in Experiments 5–7, or of experimenter administered cocaine in Experiments 1–4 was fairly short. While self-administration protocols of 2–3 weeks are standard in the field (Dickson et al. 2014; Lopez et al. 2021; Lujan et al. 2018; Roberts et al. 2018), they do not reflect human drug taking experiences which can occur over several years or decades (Kuhn et al. 2019; Spanagel 2017). Thus, we can only report on and interpret the data in terms of cocaine reward and reinforcement, rather than attempting to comprehensively model human cocaine use problems. Also, we conducted these experiments in male mice only. This was a conscious decision, as female *Nrg1 TM* HET mice exhibit a less prominent behavioural phenotype than males (Chesworth et al. 2012a; Duffy et al. 2010), and show a reduced behavioural response to THC than male *Nrg1* mutants (Boucher et al. 2007a; Long et al. 2010). Thus, it is possible there are sex-specific effects of cocaine in *Nrg1* mutants. Finally, the highest dose used in cocaine IVSA (1 mg/kg/infusion) was potentially too high [although it has been used previously: (Bird et al. 2014)] and it may have induced ceiling effects. Future work should examine lower cocaine IVSA doses.

In conclusion, this is the first report linking *Nrg1* to behaviours which may increase susceptibility for problematic cocaine use. While there are some clinical reports linking cannabis and alcohol use problems with *NRG1* mutations (Aukst Margetic et al. 2019; Han et al. 2012; Vaht et al. 2017), to date there have been no investigations into *NRG1* mutations and association with cocaine use disorder. Our data extend current knowledge by not only linking *Nrg1* with behaviours relevant to problematic cocaine use, but also identifying which cocaine-induced behaviours *Nrg1* mutation impacts on. By demonstrating extinction deficits for cocaine in *Nrg1 TM* HET mice, we demonstrate a behavioural mechanism (i.e. limiting cocaine responding) which may increase susceptibility to cocaine use in individuals carrying this mutation.

## Supplementary Information

Below is the link to the electronic supplementary material.

Supplementary file1 (DOCX 670 KB)

## Data Availability

Data is available on request from authors.

## References

[CR1] Abumaria N, Luo L, Ahn M, Liu G (2013). Magnesium supplement enhances spatial-context pattern separation and prevents fear overgeneralization. Behav Pharmacol.

[CR2] Alaghband Y, Kwapis JL, Lopez AJ, White AO, Aimiuwu OV, Al-Kachak A, Bodinayake KK, Oparaugo NC, Dang R, Astarabadi M, Matheos DP, Wood MA (2017). Distinct roles for the deacetylase domain of HDAC3 in the hippocampus and medial prefrontal cortex in the formation and extinction of memory. Neurobiol Learn Mem.

[CR3] AukstMargetic B, Peitl V, Vukasovic I, Karlovic D (2019). Neuregulin-1 is increased in schizophrenia patients with chronic cannabis abuse: Preliminary results. Schizophr Res.

[CR4] Bergstrom HC (2020). Assaying Fear Memory Discrimination and Generalization: Methods and Concepts. Curr Protoc Neurosci.

[CR5] Bernal-Gamboa R, Nieto J, Uengoer M (2018). Removing but not adding elements of a context affects generalization of instrumental responses. Learn Behav.

[CR6] Bird MK, Lohmann P, West B, Brown RM, Kirchhoff J, Raymond CR, Lawrence AJ (2014). The mGlu5 receptor regulates extinction of cocaine-driven behaviours. Drug Alcohol Depend.

[CR7] Boucher AA, Arnold JC, Duffy L, Schofield PR, Micheau J, Karl T (2007). Heterozygous neuregulin 1 mice are more sensitive to the behavioural effects of Delta9-tetrahydrocannabinol. Psychopharmacology.

[CR8] Boucher AA, Hunt GE, Karl T, Micheau J, McGregor IS, Arnold JC (2007). Heterozygous neuregulin 1 mice display greater baseline and Delta(9)-tetrahydrocannabinol-induced c-Fos expression. Neuroscience.

[CR9] Boucher AA, Hunt GE, Micheau J, Huang X, McGregor IS, Karl T, Arnold JC (2011). The schizophrenia susceptibility gene neuregulin 1 modulates tolerance to the effects of cannabinoids. Int J Neuropsychopharmacol.

[CR10] Brown RM, Short JL, Cowen MS, Ledent C, Lawrence AJ (2009). A differential role for the adenosine A2A receptor in opiate reinforcement vs opiate-seeking behavior. Neuropsychopharmacol.

[CR11] Brown RW, Maple AM, Perna MK, Sheppard AB, Cope ZA, Kostrzewa RM (2012). Schizophrenia and substance abuse comorbidity: nicotine addiction and the neonatal quinpirole model. Dev Neurosci.

[CR12] Burgdorf CE, Schierberl KC, Lee AS, Fischer DK, Van Kempen TA, Mudragel V, Huganir RL, Milner TA, Glass MJ, Rajadhyaksha AM (2017). Extinction of Contextual Cocaine Memories Requires Cav1.2 within D1R-Expressing Cells and Recruits Hippocampal Cav1.2-Dependent Signaling Mechanisms. J Neurosci.

[CR13] Busto A, Souza RP, Lobo DS, Shaikh SA, Zawertailo LA, Busto UE, Kennedy JL (2010). Cocaine and amphetamine regulated transcript (CART) gene in the comorbidity of schizophrenia with alcohol use disorders and nicotine dependence. Prog Neuropsychopharmacol Biol Psychiatry.

[CR14] Caliskan G, Stork O (2019). Hippocampal network oscillations at the interplay between innate anxiety and learned fear. Psychopharmacology.

[CR15] Cao Q, Wei Y, Deng J, Li J, Huang Y, Li Y, Zhang JC, Zhang Z, Lin S (2021). NRG1 accelerates the forgetting of fear memories and facilitates the induction of long-term depression in adult mice. Psychopharmacology.

[CR16] Cheah SY, Lawford BR, Young RM, Connor JP, Phillip Morris C, Voisey J (2014). BDNF SNPs are implicated in comorbid alcohol dependence in schizophrenia but not in alcohol-dependent patients without schizophrenia. Alcohol Alcohol.

[CR17] Chen J, Bacanu SA, Yu H, Zhao Z, Jia P, Kendler KS, Kranzler HR, Gelernter J, Farrer L, Minica C, Pool R, Milaneschi Y, Boomsma DI, Penninx BW, Tyndale RF, Ware JJ, Vink JM, Kaprio J, Munafo M, Chen X, Cotinine meta-analysis g, group Fm-a (2016). Genetic Relationship between Schizophrenia and Nicotine Dependence. Sci Rep.

[CR18] Chesworth R, Karl T (2020). Cannabidiol (CBD) reduces cocaine-environment memory in mice. Pharmacol Biochem Behav.

[CR19] Chesworth R, Downey L, Logge W, Killcross S, Karl T (2012). Cognition in female transmembrane domain neuregulin 1 mutant mice. Behav Brain Res.

[CR20] Chesworth R, Yulyaningsih E, Cappas E, Arnold J, Sainsbury A, Karl T (2012). The response of neuregulin 1 mutant mice to acute restraint stress. Neurosci Lett.

[CR21] Chesworth R, Brown RM, Kim JH, Lawrence AJ (2013). The metabotropic glutamate 5 receptor modulates extinction and reinstatement of methamphetamine-seeking in mice. PLoS ONE.

[CR22] Chesworth R, Brown RM, Kim JH, Ledent C, Lawrence AJ (2016). Adenosine 2A receptors modulate reward behaviours for methamphetamine. Addiction Biology.

[CR23] Chesworth R, Rosa-Porto R, Yao S, Karl T (2021). Sex-specific sensitivity to methamphetamine-induced schizophrenia-relevant behaviours in neuregulin 1 type III overexpressing mice. J Psychopharmacol.

[CR24] Cho Y, Ryu S, Huh I, Cho EY, Oh H, Lee YS, Lee WK, Park T, Kwon JS, Hong KS (2015). Effects of genetic variations in NRG1 on cognitive domains in patients with schizophrenia and healthy individuals. Psychiatr Genet.

[CR25] Crittenden JR, Gipson TA, Smith AC, Bowden HA, Yildirim F, Fischer KB, Yim M, Housman DE, Graybiel AM (2021). Striatal transcriptome changes linked to drug-induced repetitive behaviors. Eur J Neurosci.

[CR26] Davis H, Taylor AA, Norris C (1997). Preference for familiar humans by rats. Psychon Bull Rev.

[CR27] Dickson PE, Miller MM, Rogers TD, Blaha CD, Mittleman G (2014). Effects of adolescent nicotine exposure and withdrawal on intravenous cocaine self-administration during adulthood in male C57BL/6J mice. Addict Biol.

[CR28] Dixon L (1999). Dual diagnosis of substance abuse in schizophrenia: prevalence and impact on outcomes. Schizophr Res.

[CR29] Duffy L, Cappas E, Lai D, Boucher AA, Karl T (2010). Cognition in transmembrane domain neuregulin 1 mutant mice. Neuroscience.

[CR30] Dunsmoor JE, Paz R (2015). Fear Generalization and Anxiety: Behavioral and Neural Mechanisms. Biol Psychiat.

[CR31] Fischer DK, Rice RC, Martinez Rivera A, Donohoe M, Rajadhyaksha AM (2017). Altered reward sensitivity in female offspring of cocaine-exposed fathers. Behav Brain Res.

[CR32] Gancarz A, Jouroukhin Y, Saito A, Shevelkin A, Mueller LE, Kamiya A, Dietz DM, Pletnikov MV (2016). DISC1 signaling in cocaine addiction: Towards molecular mechanisms of co-morbidity. Neurosci Res.

[CR33] Gotra MY, Hill SK, Gershon ES, Tamminga CA, Ivleva EI, Pearlson GD, Keshavan MS, Clementz BA, McDowell JE, Buckley PF, Sweeney JA, Keedy SK (2020). Distinguishing patterns of impairment on inhibitory control and general cognitive ability among bipolar with and without psychosis, schizophrenia, and schizoaffective disorder. Schizophr Res.

[CR34] Green AI, Drake RE, Brunette MF, Noordsy DL (2007). Schizophrenia and co-occurring substance use disorder. Am J Psychiatry.

[CR35] Han S, Yang BZ, Kranzler HR, Oslin D, Anton R, Farrer LA, Gelernter J (2012). Linkage analysis followed by association show NRG1 associated with cannabis dependence in African Americans. Biol Psychiat.

[CR36] Hikida T, Morita M, Kuroiwa M, Macpherson T, Shuto T, Sotogaku N, Niwa M, Sawa A, Nishi A (2020). Adolescent psychosocial stress enhances sensitization to cocaine exposure in genetically vulnerable mice. Neurosci Res.

[CR37] Karl T, Duffy L, Scimone A, Harvey RP, Schofield PR (2007). Altered motor activity, exploration and anxiety in heterozygous neuregulin 1 mutant mice: implications for understanding schizophrenia. Genes Brain Behav.

[CR38] Khokhar JY, Dwiel LL, Henricks AM, Doucette WT, Green AI (2018). The link between schizophrenia and substance use disorder: A unifying hypothesis. Schizophr Res.

[CR39] Khoo AT, Gibson GD, Prasad AA, McNally GP (2015). Role of the striatopallidal pathway in renewal and reacquisition of alcohol seeking. Behav Neurosci.

[CR40] Khroyan TV, Yasuda D, Toll L, Polgar WE, Zaveri NT (2015). High affinity alpha3beta4 nicotinic acetylcholine receptor ligands AT-1001 and AT-1012 attenuate cocaine-induced conditioned place preference and behavioral sensitization in mice. Biochem Pharmacol.

[CR41] Kuhn BN, Kalivas PW, Bobadilla AC (2019). Understanding Addiction Using Animal Models. Front Behav Neurosci.

[CR42] Long LE, Chesworth R, Arnold JC, Karl T (2010). A follow-up study: acute behavioural effects of Delta(9)-THC in female heterozygous neuregulin 1 transmembrane domain mutant mice. Psychopharmacology.

[CR43] Long LE, Chesworth R, Huang XF, Wong A, Spiro A, McGregor IS, Arnold JC, Karl T (2012). Distinct neurobehavioural effects of cannabidiol in transmembrane domain neuregulin 1 mutant mice. PLoS ONE.

[CR44] Long LE, Chesworth R, Huang XF, McGregor IS, Arnold JC, Karl T (2013). Transmembrane domain Nrg1 mutant mice show altered susceptibility to the neurobehavioural actions of repeated THC exposure in adolescence. Int J Neuropsychopharmacol.

[CR45] Long LE, Anderson P, Frank E, Shaw A, Liu S, Huang XF, Pinault D, Karl T, O'Brien TJ, Shannon Weickert C, Jones NC (2015). Neuregulin 1 expression and electrophysiological abnormalities in the Neuregulin 1 transmembrane domain heterozygous mutant mouse. PLoS ONE.

[CR46] Lopez AJ, Johnson AR, Euston TJ, Wilson R, Nolan SO, Brady LJ, Thibeault KC, Kelly SJ, Kondev V, Melugin P, Kutlu MG, Chuang E, Lam TT, Kiraly DD, Calipari ES (2021). Cocaine self-administration induces sex-dependent protein expression in the nucleus accumbens. Commun Biol.

[CR47] Lujan MA, Castro-Zavala A, Alegre-Zurano L, Valverde O (2018). Repeated Cannabidiol treatment reduces cocaine intake and modulates neural proliferation and CB1R expression in the mouse hippocampus. Neuropharmacology.

[CR48] Malvaez M, McQuown SC, Rogge GA, Astarabadi M, Jacques V, Carreiro S, Rusche JR, Wood MA (2013). HDAC3-selective inhibitor enhances extinction of cocaine-seeking behavior in a persistent manner. Proc Natl Acad Sci USA.

[CR49] Martin AK, Robinson G, Reutens D, Mowry B (2014). Cannabis abuse and age at onset in schizophrenia patients with large, rare copy number variants. Schizophr Res.

[CR50] Menne V, Chesworth R (2020). Schizophrenia and drug addiction comorbidity: recent advances in our understanding of behavioural susceptibility and neural mechanisms. Neuroanatomy and Behaviour.

[CR51] Miller AP, Gizer IR, Fleming Iii WA, Otto JM, Deak JD, Martins JS, Bartholow BD (2019). Polygenic liability for schizophrenia predicts shifting-specific executive function deficits and tobacco use in a moderate drinking community sample. Psychiatry Res.

[CR52] Mostaid MS, Lloyd D, Liberg B, Sundram S, Pereira A, Pantelis C, Karl T, Weickert CS, Everall IP, Bousman CA (2016). Neuregulin-1 and schizophrenia in the genome-wide association study era. Neurosci Biobehav Rev.

[CR53] Nader MA, Morgan D, Gage HD, Nader SH, Calhoun TL, Buchheimer N, Ehrenkaufer R, Mach RH (2006). PET imaging of dopamine D2 receptors during chronic cocaine self-administration in monkeys. Nat Neurosci.

[CR54] Newell KA, Karl T, Huang XF (2013). A neuregulin 1 transmembrane domain mutation causes imbalanced glutamatergic and dopaminergic receptor expression in mice. Neuroscience.

[CR55] Ng E, McGirr A, Wong AH, Roder JC (2013). Using rodents to model schizophrenia and substance use comorbidity. Neurosci Biobehav Rev.

[CR56] Nguyen AT, Marquez P, Hamid A, Kieffer B, Friedman TC, Lutfy K (2012). The rewarding action of acute cocaine is reduced in beta-endorphin deficient but not in mu opioid receptor knockout mice. Eur J Pharmacol.

[CR57] Pasman JA, Verweij KJH, Gerring Z, Stringer S, Sanchez-Roige S, Treur JL, Abdellaoui A, Nivard MG, Baselmans BML, Ong JS, Ip HF, van der Zee MD, Bartels M, Day FR, Fontanillas P, Elson SL, Me Research T, de Wit H, Davis LK, MacKillop J, substance use disorders working group of the psychiatric genomics C, International Cannabis C, Derringer JL, Branje SJT, Hartman CA, Heath AC, van Lier PAC, Madden PAF, Magi R, Meeus W, Montgomery GW, Oldehinkel AJ, Pausova Z, Ramos-Quiroga JA, Paus T, Ribases M, Kaprio J, Boks MPM, Bell JT, Spector TD, Gelernter J, Boomsma DI, Martin NG, MacGregor S, Perry JRB, Palmer AA, Posthuma D, Munafo MR, Gillespie NA, Derks EM, Vink JM (2018) GWAS of lifetime cannabis use reveals new risk loci, genetic overlap with psychiatric traits, and a causal influence of schizophrenia. Nature neuroscience 21: 1161-1170 10.1038/s41593-018-0206-1 PMC6386176 30150663

[CR58] Reginsson GW, Ingason A, Euesden J, Bjornsdottir G, Olafsson S, Sigurdsson E, Oskarsson H, Tyrfingsson T, Runarsdottir V, Hansdottir I, Steinberg S, Stefansson H, Gudbjartsson DF, Thorgeirsson TE, Stefansson K (2018). Polygenic risk scores for schizophrenia and bipolar disorder associate with addiction. Addict Biol.

[CR59] Roberts AJ, Casal L, Huitron-Resendiz S, Thompson T, Tarantino LM (2018). Intravenous cocaine self-administration in a panel of inbred mouse strains differing in acute locomotor sensitivity to cocaine. Psychopharmacology.

[CR60] Sara GE, Burgess PM, Malhi GS, Whiteford HA, Hall WC (2014). Stimulant and other substance use disorders in schizophrenia: prevalence, correlates and impacts in a population sample. Aust N Z J Psychiatry.

[CR61] Skirzewski M, Cronin ME, Murphy R, Fobbs W, Kravitz AV, Buonanno A (2020) ErbB4 Null Mice Display Altered Mesocorticolimbic and Nigrostriatal Dopamine Levels as well as Deficits in Cognitive and Motivational Behaviors. eNeuro 7 10.1523/ENEURO.0395-19.2020 PMC7242816 32354758

[CR62] Sorge RE, Martin LJ, Isbester KA, Sotocinal SG, Rosen S, Tuttle AH, Wieskopf JS, Acland EL, Dokova A, Kadoura B, Leger P, Mapplebeck JC, McPhail M, Delaney A, Wigerblad G, Schumann AP, Quinn T, Frasnelli J, Svensson CI, Sternberg WF, Mogil JS (2014). Olfactory exposure to males, including men, causes stress and related analgesia in rodents. Nat Methods.

[CR63] Spanagel R (2017). Animal models of addiction. Dialogues Clin Neurosci.

[CR64] Spencer JR, Darbyshire KM, Boucher AA, Arnold JC (2012). Adolescent neuregulin 1 heterozygous mice display enhanced behavioural sensitivity to methamphetamine. Prog Neuropsychopharmacol Biol Psychiatry.

[CR65] Thanos PK, Michaelides M, Umegaki H, Volkow ND (2008). D2R DNA transfer into the nucleus accumbens attenuates cocaine self-administration in rats. Synapse.

[CR66] Vaht M, Laas K, Kiive E, Parik J, Veidebaum T, Harro J (2017). A functional neuregulin-1 gene variant and stressful life events: Effect on drug use in a longitudinal population-representative cohort study. J Psychopharmacol.

[CR67] van den Buuse M, Wischhof L, Lee RX, Martin S, Karl T (2009). Neuregulin 1 hypomorphic mutant mice: enhanced baseline locomotor activity but normal psychotropic drug-induced hyperlocomotion and prepulse inhibition regulation. Int J Neuropsychopharmacol.

[CR68] van Driel KS, Talling JC (2005). Familiarity increases consistency in animal tests. Behav Brain Res.

[CR69] Venniro M, Caprioli D, Shaham Y (2016). Animal models of drug relapse and craving: From drug priming-induced reinstatement to incubation of craving after voluntary abstinence. Prog Brain Res.

[CR70] Visini GR, Brown S, Weston-Green K, Shannon Weickert C, Chesworth R, Karl T (2022) The effects of preventative cannabidiol in a male neuregulin 1 mouse model of schizophrenia. Frontiers in cellular neuroscience 3 Nov 10.3389/fncel.2022.1010478 PMC9669370 36406747

[CR71] Volkow ND (2009). Substance use disorders in schizophrenia–clinical implications of comorbidity. Schizophr Bull.

[CR72] Walss-Bass C, Liu W, Lew DF, Villegas R, Montero P, Dassori A, Leach RJ, Almasy L, Escamilla M, Raventos H (2006). A novel missense mutation in the transmembrane domain of neuregulin 1 is associated with schizophrenia. Biol Psychiat.

[CR73] Yamamoto J, Suh J, Takeuchi D, Tonegawa S (2014). Successful execution of working memory linked to synchronized high-frequency gamma oscillations. Cell.

[CR74] Yan Y, Nitta A, Mizoguchi H, Yamada K, Nabeshima T (2006). Relapse of methamphetamine-seeking behavior in C57BL/6J mice demonstrated by a reinstatement procedure involving intravenous self-administration. Behav Brain Res.

[CR75] Yang H, Xiao W, Yang M, Wang Y, Zhang X (2021). Decreased neuregulin1beta1 in first episode and drug-naive patients with schizophrenia: Negative correlation with cognitive impairment. Psychiatry Res.

[CR76] Zai CC, Manchia M, Zai GC, Woo J, Tiwari AK, de Luca V, Kennedy JL (2018). Association study of BDNF and DRD3 genes with alcohol use disorder in Schizophrenia. Neurosci Lett.

