# Supplementary material for: Impaired extinction of operant cocaine in a genetic mouse model of schizophrenia risk
Source: Psychopharmacology (Berl). 2023 May 26;240(7):1531–46. doi: 10.1007/s00213-023-06386-8 (PMC10271887; doi:10.1007/s00213-023-06386-8)
Supplement: Supplementary file 1 — Supplementary file1 (DOCX 670 KB) [file 213_2023_6386_MOESM1_ESM.docx]

**Supplementary Figure 1A-B. Time in zones at habituation in the CPP Cocaine 10 mg/kg experiment.** Time in each zone varied across habituation (‘zone’ x ‘time’ interaction), with a preference for the right zone in WT mice at 5 min and in *Nrg1 TM* HET mice at 25 min. Data analysed with two-way RM ANOVA and Bonferroni post-hoc tests. Significant post-hoc effects of zone are indicated by hash symbols (^#^*p* < .05).

**
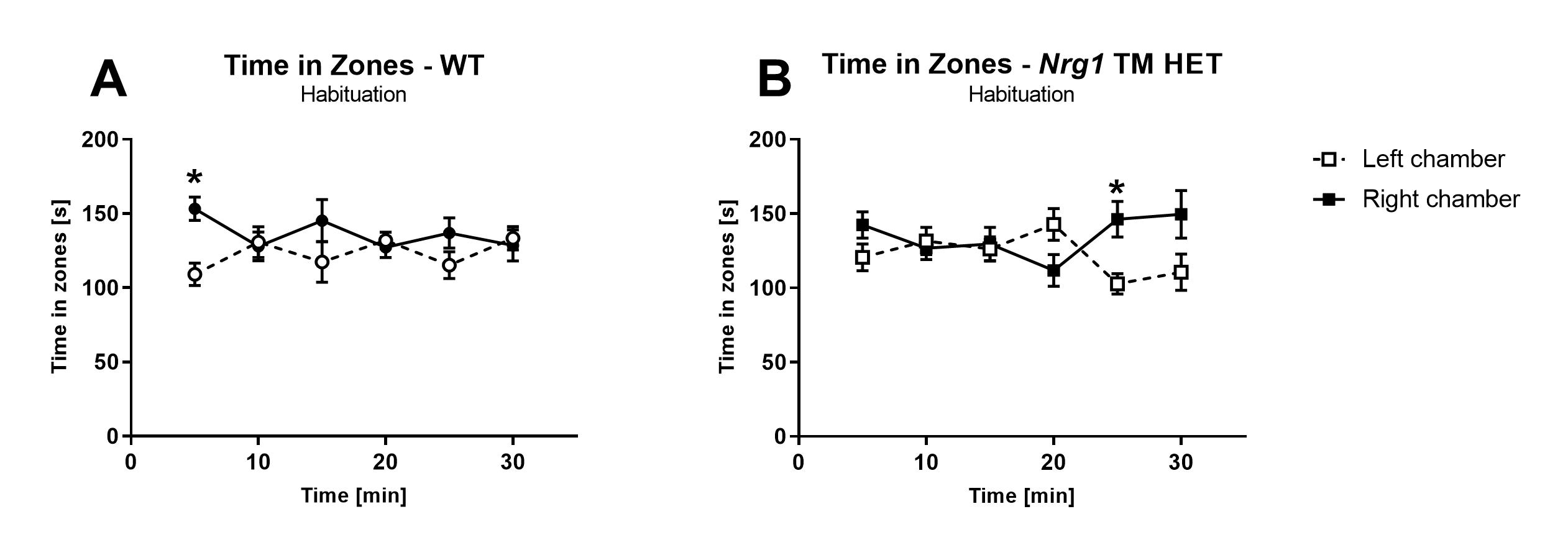
**

**Statistical Analysis:** There was a preference for the right zone in the 10 mg/kg cocaine cohort [‘zone’ F(1,16) = 5.7, *p* = .03]. However, time course data indicated only a preference for the right zone at particular time points [‘zone’ x ‘time’ interaction, F(5,80) = 4.0, *p* = .003; Supplementary Fig. 1]. Bonferroni post-hoc tests demonstrated a significant preference in the first 5-min block of the test in WT mice, and in 2nd last 5-min block in *Nrg1 TM* HET mice only - no other preferences between the zones were detected.

**Supplementary Fig 2. Operant sucrose pellet self-administration.** Active and inactive lever pressing [n] for a sucrose pellet during single lever and double lever training, in WT and *Nrg1 TM* HET mice. Mice were trained for 2 days with only the active lever (‘single lever’) and then for 5 days with the active and inactive lever present (‘double lever’). Both genotypes discriminated the active lever from the inactive lever (‘lever type’ x ‘days’ interaction, *p* < .0001; no ‘genotype’ interactions). Data analysed by three-way RM ANOVA, and presented as means ± SEM. RM effects of ‘lever type’ as indicated by ‘#’ (^###^*p* < .0001).

**
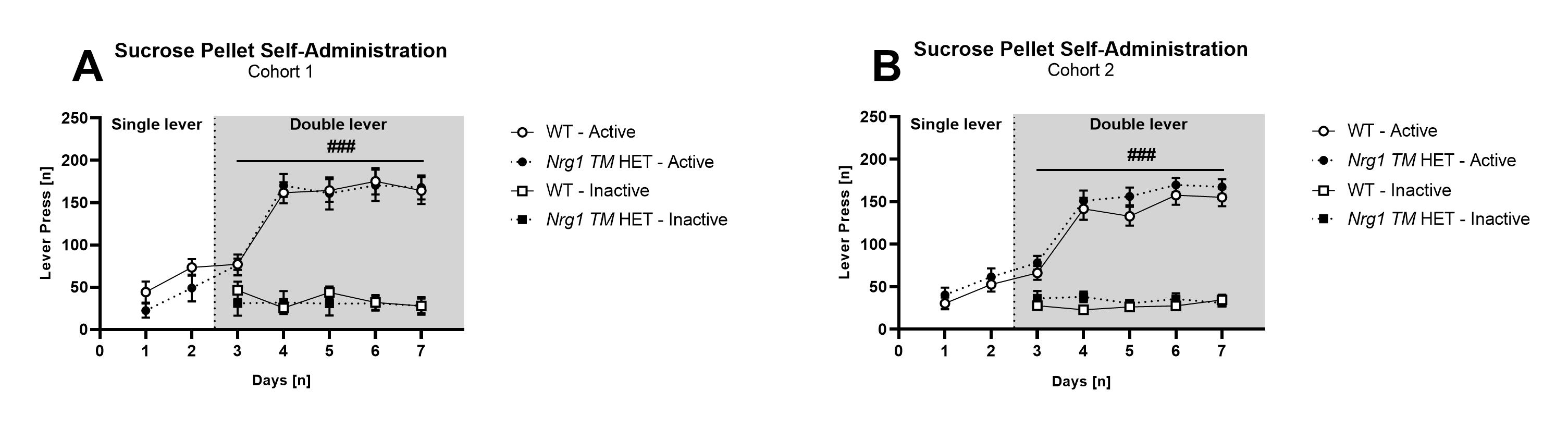
**

**Statistical Analysis:** Cohort 1 (Dose Response): During single lever training, all mice increased their responding on the active lever over the two days of training [‘days’ F(1,13) = 16.8, *p* = .001]; this was not different between the genotypes [‘genotype’ F(1,13) = 2.2, *p* = .2; no ‘day’ x ‘genotype’ interaction] (Supplementary Fig. 2A). When the inactive lever was introduced, both genotypes preferred the active lever [‘lever type’ F(1,13) = 160.8, *p* < .0001; no interaction with ‘genotype’], and discrimination for the active lever improved across the 5 days of double lever training [‘lever type’ x ‘days’ interaction: F(4,52) = 26.4, *p* < .0001; no ‘lever type’ x ‘days’ x ‘genotype’ interaction]. There were no genotype differences in overall lever responding during double lever training [‘genotype’ F(1,13) = .03, *p* = .9] (Supplementary Fig. 2A).

Cohort 2 (Extinction & Reinstatement): All mice increased their responding on the active lever during single lever training [‘days’ F(1,37) = 18.1, *p* < .0001]; this was not different between the genotypes [‘genotype’ F(1,37) = 1.2, *p* = .3; no ‘day’ x ‘genotype’ interaction]. Both genotypes preferred the active to the inactive lever [‘lever type’ F(1,37) = 213.0, *p* < .0001; no ‘genotype’ interaction], and active lever discrimination improved during double lever training [‘lever type’ x ‘days’ interaction: F(4,148) = 56.1, *p* < .0001]. No genotype differences were detected during double lever training [‘genotype’: F(1,37) = 2.2, *p* = .1; no ‘lever type’ x ‘days’ x ‘genotype’ interaction] (Supplementary Fig. 2B).

**Supplementary Figure 3. Timecourse of lever pressing during the first and final days of cocaine and sucrose extinction in WT and *Nrg1 TM* HET mice.** Timecourse of active and inactive lever pressing for A) the first day of cocaine extinction and B) the last day of cocaine extinction, and the C) the first day of sucrose extinction and D) the last day of sucrose extinction in WT and *Nrg1 TM* HET mice. Data analysed with four-way RM ANOVA and presented as means ± SEM. Post-hoc effects of genotype on the active lever are indicated by asterisks (**p* < .05), genotype effects on the inactive lever are indicated by ‘^’ (^*p* < .05, ^^*p* < .01).

**
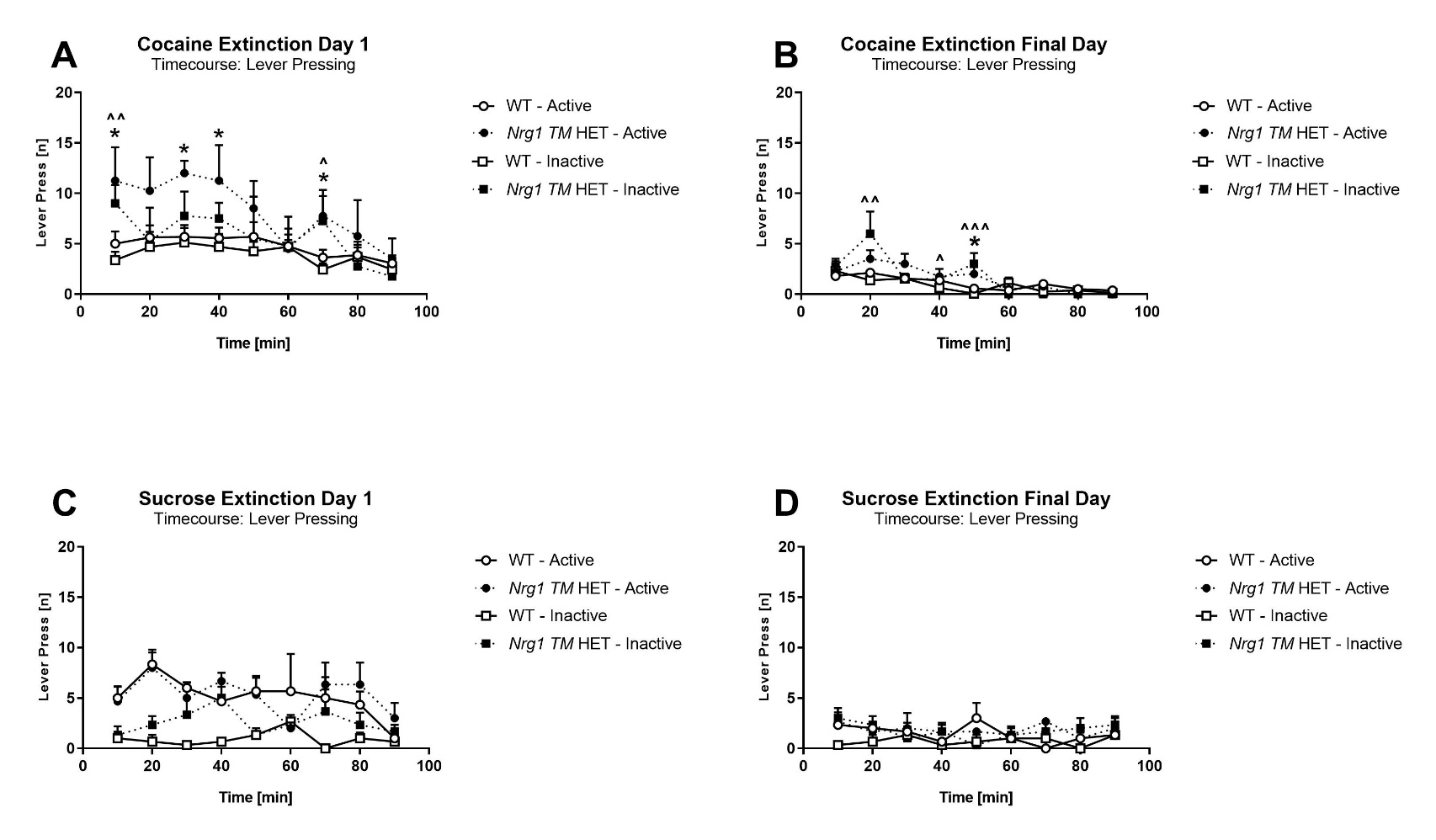
**

**Supplementary Figure 4. Total Active and Inactive Lever Press during Extinction and Cue-Induced Reinstatement for Cocaine and Sucrose.** Data analysed with three-way RM ANOVA and presented as means ± SEM. There was a main effect of ‘genotype’ in B). **Abbreviations: EXT FINAL: final day of extinction.**


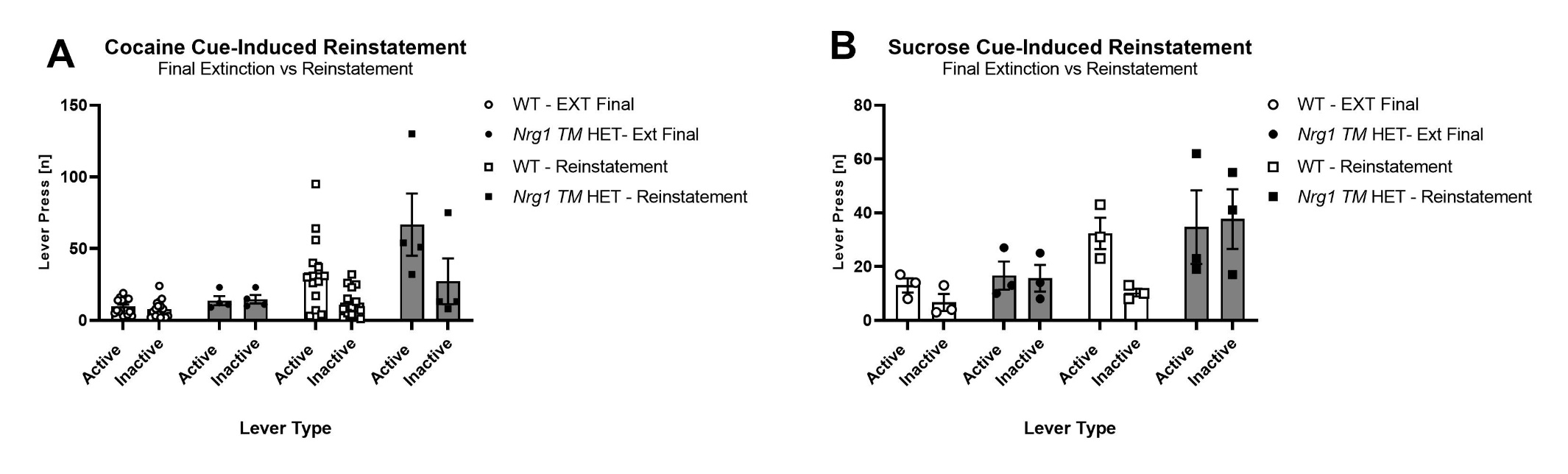


**Supplementary Table 1. Timeline for Conditioned Place Preference Experiments 1-4.** During conditioning sessions, mice were administered saline or 5 mg/kg cocaine (Experiment 1), 10 mg/kg (Experiment 2), 20 mg/kg (Experiment 3) or 30 mg/kg (Experiment 4). Abbreviations: CPP: Conditioned Place Preference.

| **Day / Time** | **Test** |
| --- | --- |
| Day 1: 2pm | CPP Habituation |
| Day 2: 9am | CPP Saline Conditioning |
| Day 2: 2pm | CPP Cocaine Conditioning |
| Day 3: 9am | CPP Saline Conditioning |
| Day 3: 2pm | CPP Cocaine Conditioning |
| Day 4: 9am | CPP Saline Conditioning |
| Day 4: 2pm | CPP Cocaine Conditioning |
| Day 5: 9am | CPP Saline Conditioning |
| Day 5: 2pm | CPP Cocaine Conditioning |
| Day 6: 9am | CPP Test |

**Supplementary Table 2. Timeline for Cocaine Intravenous Self-Administration Experiment 5.** Mice were trained to self-administer sucrose pellets prior to jugular vein surgery. Mice were tested with 3 doses of cocaine under FR2 and progressive ratio conditions (FR2 and PR completed prior to changing dose): 0.5, 0.1 then 1 mg/kg cocaine. Abbreviations: FR2: fixed ratio 2; IVSA: intravenous self-administration.

| **Day** | **Test** |
| --- | --- |
| Day 1-8 | Sucrose pellet training |
| Day 9-11 | Free food prior to surgery |
| Day 12-19 | Surgery and recovery |
| Day 20-23 | Cocaine IVSA FR2 0.5 mg/kg - acquisition (food restriction easing) |
| Day 24-28 | Cocaine IVSA FR2 0.5 mg/kg - Stable FR2 |
| Day 29 | Cocaine IVSA FR2 0.5 mg/kg - Progressive Ratio |
| Day 30 | Day off |
| Day 31 - 35 | Cocaine IVSA FR2 0.1 mg/kg - Stable FR2 |
| Day 36 | Cocaine IVSA FR2 0.1 mg/kg - Progressive Ratio |
| Day 37 | Day off |
| Day 38-42 | Cocaine IVSA FR2 1 mg/kg - Stable FR2 |
| Day 43 | Cocaine IVSA FR2 1 mg/kg - Progressive Ratio |

**Supplementary Table 3. Timeline for Cocaine Intravenous Extinction and Reinstatement Experiment 6.** Mice were trained to self-administer sucrose pellets prior to jugular vein surgery. Mice were assessed for cocaine self-administration (0.5 mg/kg/infusion) prior to up to 10 days of extinction (no cocaine or cocaine-cues present). If mice met extinction criteria within 10 days, they were tested on the subsequent day for cue-induced reinstatement. Abbreviations: FR2: fixed ratio 2; IVSA: intravenous self-administration.

| **Day** | **Test** |
| --- | --- |
| Day 1-8 | Sucrose pellet training |
| Day 9-11 | Free food prior to surgery |
| Day 12-19 | Surgery and recovery |
| Day 20-23 | Cocaine IVSA FR2 0.5 mg/kg - acquisition (food restriction easing) |
| Day 24-33 | Cocaine IVSA FR2 0.5 mg/kg - Stable FR2 |
| Day 34-43 | Extinction and Reinstatement (reinstatement conducted the day after which mice met extinction criteria) |

**Supplementary Table 4. Timeline for Sucrose Self-Administration Experiment 7.** Mice were trained to self-administer 10% sucrose for 15 days, prior to up to 10 days of extinction (no sucrose or sucrose-cues present). If mice met extinction criteria within 10 days, they were tested on the subsequent day for cue-induced reinstatement. Abbreviations: FR2: fixed ratio 2.

| **Day** | **Test** |
| --- | --- |
| Day 1-15 | Sucrose self-administration |
| Day 16-25 | Extinction and Reinstatement (reinstatement conducted the day after which mice met extinction criteria) |

**Supplementary Table 5. Time in zones at habituation, for each dose tested.** Data analysed with one-way ANOVA. Main effects of ‘zone’ are indicated by hash symbols (^##^*p* < .01). Abbreviations: CPP: conditioned place preference.

|  | **WT** | | ***Nrg1 TM* HET** | |  |
| --- | --- | --- | --- | --- | --- |
| **Experiment** | **Time in Left zone**  **(white walls)** | **Time in Right zone (white walls with black spots)** | **Time in Left zone**  **(white walls)** | **Time in Right zone (white walls with black spots)** | **Main effect of zone** |
| CPP 5 mg/kg cocaine | 841.3 ± 26.4 | 758.3 ± 28.7 | 773.9 ± 32.9 | 784.9 ± 27.3 | F(1,26) = .8, *p* = .4 |
| CPP 10 mg/kg cocaine | 737.7 ± 18.5 | 819.1 ± 20.9 | 734.4 ± 21.2 | 806.4 ± 30.9 | F(1,16) = 6.6, *p* = .02 ## |
| CPP 20 mg/kg cocaine | 756.9 ± 27.2 | 879.3 ± 31.5 | 748.6 ± 38.8 | 768.5 ± 23.1 | F(1,23) = 28, *p* = .1 |
| CPP 30 mg/kg cocaine | 796.9 ± 24.2 | 759.4 ± 33.5 | 777.4 ± 33.9 | 763.3 ± 27.8 | F(1,27) = .4, *p* = .5 |
